# Supplementary material for: Synergistic sequence contributions bias glycation outcomes
Source: Nat Commun. 2021 Jun 3;12:3316. doi: 10.1038/s41467-021-23625-8 (PMC8175500; doi:10.1038/s41467-021-23625-8)
Supplement: Supplementary file 1 — Supplementary Information [file 41467_2021_23625_MOESM1_ESM.pdf]

Synergistic Sequence Contributions Bias Glycation Outcomes

Joseph M. McEwen, Sasha Fraser, Alexxandra L. Sosa Guir, Jaydev Dave, and Rebecca A. Scheck*

Tufts University, Department of Chemistry, 62 Talbot Ave, Medford MA
*rebecca.scheck@tufts.edu

Supplementary Information

Supplementary Materials and Methods

General Materials	3
Peptide Synthesis	3
Peptide Purification	3
MALDI Mass Spectrometry	3
Liquid Chromatography and Mass Spectrometry	3
LC-MS Analysis and Quantification (**Eq. 1**)	4
General Protocol for Glycation of Peptides	4
Library Design and Synthesis	5
Library Screening with α-MGH-1 Abs	5
Library Screening using Impaired Proteolysis to Trypsin	6
MGO Treatment for ANP-Linked Resin-Bound Peptides	6
Preparation of MGH-1-Modified Peptide **1** ($\mathbf{1}^{MGH\text{-}1}$)	7
Preparation of CEA-Modified Peptide **1** ($\mathbf{1}^{CEA}$)	7
NMR Acquisition	7
General Protocol for Dilution Experiments	7
Chemical Derivatization of AGEs using Boronic Acids	8
Chemical Derivatization of AGEs by EDC/HOBt Coupling	8
Mammalian cell culture	8
Plasmids for Protein Expression	8
Cellular MGO Assays	9
Immunoprecipitation Protocol	10
Western Blotting	10

Supplementary Figures and Tables

**Supplementary Fig. 1**	Primary Sequence Influences Glycation Outcomes	11
**Supplementary Fig. 2**	An [M+12] AGE is Exclusive to N-terminal Proline	12
**Supplementary Fig. 3**	Detection of MGH-1-Modified Peptides on Resin	13
**Supplementary Fig. 4**	Optimization of Library Screening Conditions with α-MGH-1 Antibodies	14
**Supplementary Fig. 5**	Single Bead Sequencing using LC-MS/MS	15
**Supplementary Table 1**	Hit Sequences Identified	16
**Supplementary Fig. 6**	Arg-Containing Sequences from Ovalbumin Do Not Match Consensus Motif	17
**Supplementary Fig. 7**	Library Screening using Impaired Proteolysis by Trypsin	18
**Supplementary Table 2**	Non-Glycated Sequences Possess Dense Negative Charge	19
**Supplementary Fig. 8**	Validation of Library-Derived "Hit" and "Non-Hit" Peptides.	20
**Supplementary Fig. 9**	Point Variants of Peptide **1**	21
**Supplementary Fig. 10**	Point Variants of Peptide **2**	22
**Supplementary Fig. 11**	Analysis of Glycation on Resin	23
**Supplementary Fig. 12**	Mapping the Positional Contribution of Tyr	24
**Supplementary Fig. 13**	Scrambled Variants Suggest Cooperative Interactions Influence Glycation Outcomes	25
**Supplementary Fig. 14**	Extended Glycation Reactions Reveal MGH-1 as a Predominant AGE	26
**Supplementary Fig. 15**	Chemical Derivatization of Peptide **1** [M+72] Adducts	27
**Supplementary Fig. 16**	Characterization of Peptide $\mathbf{1}^{\text{MGH-1}}$ by NMR	28
**Supplementary Fig. 17**	Characterization of Peptide $\mathbf{1}^{\text{CEA}}$ by NMR	29
**Supplementary Fig. 18**	Chemical Derivatization of Peptide $\mathbf{1}^{\text{CEA}}$	30
**Supplementary Fig. 19**	MGH-1 is Directly Converted to CEA	31
**Supplementary Fig. 20**	A Putative Peptide 1 MGH-3 Adduct Rapidly Produces CEA	32
**Supplementary Fig. 21**	Companion to Main Text Figure 6	33
**Supplementary Fig. 22**	Evaluating Glycation at Different pH	34
**Supplementary Fig. 23**	Chlorinated Peptide **1** Variants Accelerate MGH-1 Formation	35
**Supplementary Fig. 24**	Peptide Sequence can Extend AGE Lifetimes	36
**Supplementary Fig. 25**	Expression and Enrichment of GFP-**1**, GFP-$\mathbf{1}^{\text{Ala}}$, and GFP-**3** from HEK-293T cells	37
**Supplementary Fig. 26**	MGO treatment of GFP-**1**, GFP-$\mathbf{1}^{\text{Ala}}$, and GFP-**3** in HEK-293T cells	38
**Supplementary Fig. 27**	Companion to Main Text Figure 7	39
**Supplementary Fig. 28**	Uncropped Immunoblots	40

Supplementary References 41

General Materials. All chemical reagents and solvents were of analytical grade, obtained from commercial suppliers and used without further purification unless otherwise noted. Methylglyoxal (MGO) (40% w/v in water) was purchased from MilliporeSigma (M0252). Any commercially-available peptides were purchased from AnaSpec Inc., including MOG (res. 8-21; AS-62369), Mgp100 (res. 25-33; AS-64752), MAGE-3 (res. 271-279; AS-61355), HER2 (res. 420- 429; AS-64837), β-Amyloid, (res. 1-10; AS-64478), TfR Targeting Peptide (AS-64689), and VIP (res. 1-12; AS-24217). HEK-293T cells were purchased from ATCC. The rat α-GFP antibody (3H9) and GFP-Trap® kit (GTAK-20) were purchased from Chromotek. The mouse α-MGH-1 primary antibody was purchased from Cell BioLabs, Inc. (STA-011). The mouse α-GAPDH primary antibody was purchased from MilliporeSigma (G8795). Alkaline phosphatase-conjugated goat α-mouse secondary antibody was purchased from Abcam (AB7069). HRP-linked α-mouse secondary antibody (7076V), and α-rat secondary antibody (7077S) were purchased from Cell Signaling Technology. Sequencing grade trypsin (V5111), streptavidin conjugated to alkaline phosphatase (V5591) and the BCIP/NBT reagents (S3771) were purchased from Promega. Dimethyl Sulfoxide-d_6 for NMR analysis was purchased from Cambridge Isotope Laboratories, Inc (DLM-10-10X0.75). Fmoc-L-Tyr(3-Cl)-OH was purchased from Chem-Impex International, Inc. (15121). Fmoc-L-Tyr(3,5-Cl_2)-OH was purchased from WuXi AppTec Inc. (LN03245735). All other Fmoc-protected amino acid monomers were purchased from ChemPep Inc. or Advanced ChemTech Inc.

Peptide Synthesis. Peptides were prepared using standard Fmoc-based solid phase peptide synthesis on Trityl chloride resin (200 mesh, 1.6 mmol/g loading, Novabiochem®) or Fmoc-Ala-Wang resin (100-200 mesh, 0.6 mmol/g loading, CreoSalus Inc.), typically on a 100 μmol scale in a 10 mL polypropylene fritted syringe. For Trityl chloride resin only, C-terminal amino-acids (5 equiv., 500 μmol) were coupled overnight in a solution of 10 equiv. (1 mmol) N,N-diisopropylethylamine (DIEA) in 3 mL dimethylformamide (DMF). The next day, the resin was washed and capped with a solution (3 mL) of DMF:MeOH:DIEA (17:2:1) for 1 hour before further deprotection & coupling steps. C-terminal coupling and resin capping was not required when using Fmoc-Ala-Wang resin. In either case, subsequent amino acids were added after Fmoc-deprotection (4 mL of 20% piperidine in DMF, 2 x 15 min), and washing with DMF (4 mL, 4 x 1 min). Couplings were accomplished by incubation of 5 equiv. of amino acid, relative to resin loading, with O-(benzotriazol-1-yl)-N,N,N′,N′-tetramethyluronium hexafluorophosphate (HBTU, 5 equiv.) and DIEA (10 equiv.) for 1 hour in a total volume of 2-3 mL of DMF. These were followed by cycles of Fmoc-deprotection and washing with DMF prior to the subsequent coupling step. N-terminal acetylation was achieved by incubation with a solution of acetic anhydride (1.5 equiv.) with DIEA (3 equiv.) for 2 hours in 3 mL DMF. The side chain protecting groups used were as follows: Arg(Pbf), Asn(Trt), Asp(tBu), Cys(Trt), Gln(Trt), Glu(tBu), His(Trt), Ser(Trt), Thr(tBu), and Tyr(tBu). Side-chain acid-deprotection and peptide cleavage was conducted with trifluoroacetic acid (TFA), triisopropylsilane (TIPS) and water (95:2.5:2.5) in 4 mL of acid solution per 100 mg resin. The acid solution was then concentrated under air flow and re-dissolved in 1-3 mL of a water/acetonitrile mixture, which varied due to peptide solubility, prior to purification.

Peptide Purification. Peptides were purified on semi-preparative scale using an Agilent ZORBAX SB-C18 column (9.4 x 250 mm, 5 μm particle size) on an Agilent 1260 Infinity LC system with a water/acetonitrile mobile phase containing 0.1% TFA. Crude peptide solutions (50-100 μL) were injected onto the column and eluted using a standard purification gradient of 5-45% acetonitrile in water over 20 minutes at a flow rate of 3.0 mL/min. Eluting peaks were monitored by absorbance at 215 nm and 280 nm and were collected using an automated fraction collector. Collected fractions were characterized using a Bruker Microflex matrix-assisted laser desorption/ionization time-of-flight (MALDI-TOF) mass spectrometer and/or an Agilent 6530 quadrupole time-of-flight (Q-TOF) mass spectrometer (see below for details) to assess peptide purity. Pure fractions were combined and lyophilized, and then each was prepared as a stock peptide solution, 20 mM in DMF or 10 mM in 50% DMF/water, depending on solubility.

MALDI Mass Spectrometry. Matrix Assisted Laser Desorption Ionization-Time of Flight (MALDI-TOF) mass spectra were obtained on a Bruker Microflex MALDI-TOF. Samples were co-crystallized using saturated solutions of α-cyano-4-hydroxycinnamic acid in 50% acetonitrile, 50% water with 0.1% trifluoroacetic acid onto ground steel plates.

Liquid Chromatography-Mass Spectrometry. Reversed phase liquid chromatography and mass spectrometry (LC-MS) analysis was carried out using an Agilent 1260 Infinity LC system coupled with Agilent 6530 Accurate Mass Q-TOF. Peptide reaction mixtures were injected onto an AdvanceBio Peptide 2.7 μm column (2.1 x 150 mm, Agilent), and eluted with a binary mobile phase of water with 0.1% formic acid (A) and acetonitrile with 0.1% formic acid (B). The elution method was as follows: isocratic 5% B from 0-1.75 min (0.400 mL/min); a gradient change of 5% B to 40% B from 1.75-16.00 min (0.400 mL/min); and a gradient change of 40% B to 100% B from 16.00-20.00 min with column heating at 55 °C. Mass spectrometry was accomplished with an electrospray ionization (ESI) source in the positive mode and spectra in the range of 300-3000 (m/z) were collected at a rate of 5 scans/sec from 1.75-20.00 min of the chromatography method. MS acquisition was achieved using the parameters: ESI capillary voltage, 4000 V; fragmentor, 150 V; gas temperature, 325 °C; gas rate, 12.5 L/min; nebulizer, 40 psig. When relevant, precursor ions were automatically selected for tandem mass spectrometry (collision induced dissociation) to generate MS/MS fragmentation spectra. Automated selection was based on both absolute (>200 counts) and relative (>0.01% of total counts) per cycle. MS/MS spectra were acquired at a rate of 3 scans/sec using a ramped collision energy model with a slope of 3.6 V and an offset of -4.8 V, a process that increases collision energy based on precursor ion drift time.

LC-MS Analysis and Quantification. Analysis of mass spectrometry data was carried out using Agilent MassHunter BioConfirm Qualitative Analysis software and PEAKS Studio (v. 7.5) software. MS data was quantified using the MassHunter Molecular Feature Extractor, which reports cumulative ion counts (MS) as 'volumes' observed for any and all charge states associated with a particular ion. As peptides and their modified counterparts can ionize differently (eg. different charge states or different salt adducts) this method provides a more robust measure than comparing only a single charge state. For each peptide sample, compound lists were generated for each replicate of MGO treatments. Quantification was carried out by dividing the AGE adduct(s) volume by the total volume of both modified and unmodified peptide (Equation 1). This quantification approach allows for a robust comparison of glycation extents across different AGEs on different peptide substrates, even though each may exhibit some variation in ionization efficiency[1]. Additionally, absolute counts of peptides that were not treated with MGO were first evaluated to confirm that roughly similar levels of ionization were observed at the same known concentration. Retention time (RT) was used to identify discrete isomers with degenerate masses.

AGE adducts for AcLESRHYA + MGO using standard glycating conditions and LC-MS acquisition

Exact Mass (Da)	RT (min)	Volume (au)	Δ mass (Da)	mod %
916.441803	9.188	1469866	0.00	NA
970.449242	9.807	162863	54.01	7.04 %
988.461967	9.319	567643	72.02	24.53 %
1060.479858	9.416	45312	144.04	1.96 %
1060.480861	10.129	29184	144.04	1.23 %
1060.483450	9.727	23949	144.04	1.03 %
		2298817		35.79%

Equation 1: $$glycation\ \% = \frac{volume\ of\ AGE\ adduct(s)}{total\ volume\ (unmodified{+}modified\ peptide)}$$

For tandem mass spectrometry, the collected scans were combined and reported for each identified precursor ion for each compound that triggered MS/MS acquisition. ProteinProspector (UCSF), an online proteomics tool, was used to generate a list of expected b and y ions for modified and unmodified peptides, which was used to assign the site of modification (See **Supplementary Figs**. **2**, **5**, & **18**). PEAKS studio (v 7.5) software was used for the *de novo* sequencing of the raw MS/MS data generated from peptides that were cleaved from single beads selected during library screening.

General Protocol for Glycation of Peptides. In general, glycation reactions for peptides in solution were performed at a 50 μL scale in 200 μL Eppendorf tubes. Commercially-available peptides (see **Supplementary Fig. 1**) were purchased as lyophilized powders and were reconstituted in 1:1 DMF:water to final stock peptide concentrations of 10 mM. Synthetic peptides purified in our laboratory were prepared as stock solutions that were 20 mM in DMF or

10 mM in 50% DMF/water, depending on solubility. MGO stocks were prepared by dilution of 15.3 μL of 40% w/v solution into 10 mL of ultrapure water (10 mM) and were stored at 4 °C for up to two weeks. To perform *in vitro* glycation, to 30 μL of ultrapure water was added 5 μL of a 10 mM stock peptide solution in 50% DMF/water, 10 μL of 100 mM phosphate buffered saline (PBS) at pH 7.3, and, lastly, 5 μL of a 10 mM MGO stock in water. The final concentrations were 1 mM peptide and 1 mM MGO in 20 mM PBS with 5% DMF co-solvent. MGO was always added in the final step to prevent any high concentration MGO exposure, and the glycation of each peptide was assessed individually. Tubes were capped, briefly spun in a benchtop microcentrifuge and incubated in a 37 °C water bath, typically for 3 h and in some cases up to 4 weeks. After incubation, peptide samples were diluted (1:100) into 5 mM Tris Buffer pH 7.3 to quench the reaction, unless otherwise noted, and subsequently subjected to LC-MS analysis.

Library Design and Synthesis. A one-bead one-compound peptide library was designed based on a sequence derived from human serum albumin (-LLVRYTK-) that we,[2] and others,[3] have found to be glycated by MGO *in vitro*. Our previous work revealed this site to be the most reactive in HSA, exclusively producing only [M+54] and [M+72] adducts. Although our past work has indicated that Lys is far less reactive towards MGO than Arg,[2] and we have found only nominal evidence of N-terminal glycation, we chose to replace Lys with Ala, and cap all N-termini to avoid potential competition from other nucleophiles. The library scaffold consisted of a C-terminal tetra *β*-alanine spacer, a central scaffold sequence of LXXRXXA, an amino-hexanoic acid (Ahx) linker, and, finally, an N-terminal cap (either biotin or acetyl). The four variable positions were randomized with 14 different amino acids: Ala, Asn, Asp, Gln, Glu, Gly, His, Leu, Phe, Ser, Thr, Trp, Tyr, and Val. All of the canonical amino acids were introduced at these positions, except for those with degenerate masses (Ile), prone to oxidation (Cys & Met), susceptible to competition with the central arginine (Arg & Lys), or that exhibit potentially confounding structural effects (Pro). The resulting combinatorial library of peptides thus contained 38,416-members with the full-length sequence being (Ac/Bio)-Ahx-LXXRXXA-$A_{\beta}A_{\beta}A_{\beta}A_{\beta}$ conjugated C-terminally to the resin via a *p*-hydroxymethyl-benzoic acid (HMBA) linker. The HMBA linker allows for side chain deprotection using standard trifluoracetic acid conditions, aqueous treatments of resin-bound peptides, and hydroxide base-mediated cleavage prior to analysis.

Standard solid phase peptide chemistry was used to achieve the library synthesis on the aqueous compatible ChemMatrix® resin (100-200 mesh, 0.50 mmol/g loading, PCAS BioMatrix, Inc., 1040378). To begin, the resin (~500,000 beads, 250 μmol) was swelled in DMF (10 mL), and placed on a shaker for approximately 45 min, in a 20 mL polypropylene fritted syringe. To couple the first amino acid, 5 equiv. (1.25 mmol) of Fmoc-β-Ala-OH was dissolved in DMF and was pre-activated with 5 equiv. (1.25 mmol) of diisopropylcarbodiimide (DIC). Subsequently, 0.1 equiv. (2.5 μmol) of 4-dimethylaminopyridine (DMAP) was added to the pre-activated amino acid, and the resulting solution was incubated with the swelled resin overnight. Excess reagent was removed by washing with approximately 10 mL DMF for a total of 5 cycles of brief shaking and draining. This coupling step was performed twice to ensure complete loading of the first amino acid to the HMBA linker. Subsequent additions of amino acids were achieved using iterative cycles of Fmoc-deprotection and coupling, as described above, though coupling steps were extended to 1.5 hours during randomization steps. To introduce randomized amino acids at four variable positions, standard split and pool methods were used. For these steps, the resin was split into 15 scintillation vials by capping a 20 mL syringe, adding 15 mL of DMF and placing 1 mL of slurry into each vial (estimated to be roughly 0.1 mmol of peptide. Following completion of the peptide sequence, an amino-hexanoic acid spacer was coupled using standard coupling protocols, as described above. The final synthesis step included either the addition of an N-terminal acetyl or biotin to cap the N-terminus. N-terminal acetylation was achieved by incubation with a solution of acetic anhydride (1.5 equiv.) with DIEA (3 equiv.) for 2 hours in 10 mL DMF. For N-terminal biotinylation, beads were incubated with 5 equiv. (1.25 mmol) of D(+)-Biotin (MilliporeSigma, 8.51209), 5 equiv. (1.25 mmol) of HBTU and 10 equiv. (2.50 mmol) DIEA in a 50% DMSO/DMF solution (10 mL). After synthesis completion, beads were washed extensively with dichloromethane (DCM), air dried, and stored under vacuum desiccation.

Library Screening with α-MGH-1 Antibodies. As the library size is approximately 40,000 unique sequences, the following screening protocol was designed to oversample the library 3X within each replicate, and was completed in triplicate to ensure proper library sampling[4]. To begin, 120 mg of N-terminally acetylated peptide library (~120,000 beads) were side chain deprotected using a solution of TFA:TIPS:H_2O (95:2.5:2.5) in a total volume of 20

mL, shaking in a polypropylene fritted syringe for 5 hours. Beads were then washed 5X with water and allowed to equilibrate in DI water overnight. The next day, beads were washed 5X with 20 mM PBS at pH 7.3 and then exposed to 0.5 mM MGO for 3 h at 37 °C. Following incubation, excess MGO was removed via washing (3X) with 20 mM Tris buffered with 150 mM NaCl and containing 0.1% Tween (TBST). The resulting MGO-modified beads were blocked using a 1% solution of bovine serum albumin (BSA) in TBST for 2 h at room temperature. Next, the blocked beads were incubated with a 1:1000 dilution of α-MGH-1 antibody (Cell BioLabs, Inc., STA-011) for 18-20 h. This antibody was developed using MGH-modified ovalbumin[5]. It has been reported to be specific for MGH-1, based on a competition assay using several AGE-modified antigens, and has been validated for the detection of MGH-1 using synthetic MGH-modified immunogens[5,6]. After incubation with primary antibody, the beads were washed 5X with TBST and then exposed to a 1:1000 dilution of α-mouse secondary antibody (Abcam, AB7069) conjugated to alkaline phosphatase for 4 h. After five washes with TBST to remove excess secondary antibody, beads were equilibrated in alkaline phosphatase buffer (100 mM Tris-HCl, 150 mM NaCl, 1 mM $MgCl_2$ at pH 9.0) for 1 h. Following equilibration, the beads were exposed to color developing reagents 5-bromo-4-chloro-3-indolyl phosphate and nitro blue tetrazolium (BCIP/NBT, Promega, S3771) and were transferred to a petri dish and imaged under a microscope equipped with a camera (Leica DMi8). Dark purple beads were manually selected and cleaved using 50 μL of 100 mM sodium hydroxide per bead. The resulting single-bead cleavage mixtures were diluted 50% in 100 mM hydrochloric acid to neutralize the solution, filtered using fritted microcentrifuge spin columns and subjected directly to sequencing by LC-MS/MS. As a result, a total of 75 beads were selected out of a cumulative 360,000 possible beads.

Library Screening using Impaired Proteolysis by Trypsin. As the library size is approximately 40,000 unique sequences, the following screening protocol was designed to oversample the library 3X within each replicate, and was completed in triplicate to ensure proper library sampling[4]. 120 mg of N-terminally biotinylated peptide library (~120,000 beads) were side chain deprotected using a solution of TFA:TIPS:H_2O (95:2.5:2.5) in a total volume of 20 mL, shaking in a polypropylene fritted syringe for 5 hours. Beads were then washed 5X with water and allowed to equilibrate in DI water overnight. The next day, beads were washed 5X with 20 mM PBS at pH 7.3 and then exposed to 0.5 mM MGO for 3 h at 37 °C. Following incubation, excess MGO was removed via washing (3X) with 20 mM Tris buffered with 150 mM NaCl and containing 0.1% Tween (TBST). The resulting MGO-modified beads were washed 3X in PBS and exposed to sequencing grade modified trypsin (Promega, V5111) for 48 h. As glycated arginine residues are resistant to trypsin activity, this treatment resulted in truncation only for unmodified beads, resulting in the removal of the N-terminal biotin tag for any sequences that did not react with MGO. After trypsin exposure, beads were washed 3X in TBST and blocked for 2 h using a solution of 0.1% BSA in TBST. Next, beads were incubated with a 1:1000 dilution of streptavidin alkaline phosphatase (Promega, V5591) in 0.1% BSA in TBST. The beads were then equilibrated in alkaline phosphatase buffer (100 mM Tris-HCl, 150 mM NaCl, 1 mM $MgCl_2$ at pH 9.0) for 1 h. Following equilibration, the beads were exposed to color developing reagents BCIP and NBT (Promega, S3771), transferred to a petri dish and imaged under a microscope equipped with a camera (Leica DMi8). Dark purple beads were manually selected and cleaved using 50 μL of 100 mM sodium hydroxide. The resulting single-bead cleavage mixtures were diluted 50% in 100 mM hydrochloric acid to neutralize the solution, filtered using fritted microcentrifuge spin columns and subjected directly to sequencing by LC-MS/MS. As a result, a total of 30 (20 positive, purple; 10 negative, colorless) beads were selected out of a cumulative 360,000 possible beads.

MGO Treatments for ANP-Linked Resin-Bound Peptides. To compare the performance of the top hit selected from the library (LESRHYA, peptide **1**) and a control sequence derived from human serum albumin screening (LLVRYTA, peptide **4**) while still attached to resin, it was necessary to find an alternative to the strongly basic cleavage conditions used to liberate peptides from the HMBA-linked resin. To do so, sequence matching peptide **1** and **4** were synthesized on ChemMatrix resin in a similar format as described in the library design section. However, for these studies, a photocleavable amino-nitrophenyl-propionic acid linker (ANP) was incorporated in place of the third β-alanine spacer, and two 2-PEG spacers were used after ANP resulting in the following sequence: Ac-LXXRXXA-(2-PEG)$_2$-**ANP**-$(A_\beta)_2$. To evaluate differences in glycation between these two sequences, resin charged with either peptide **1** or peptide **4** (~5 mg) were side chain deprotected and equilibrated in a 20 mM PBS solution at pH 7.3. Following equilibration, they were treated with 0.5 mM MGO for 3 h at 37 °C in 20 mM PBS at pH 7.3. After MGO treatment, beads were washed 5X with 50 mM Tris buffer at pH 7.3 and allowed to incubate in this buffer for 24 h at room temperature to mimic the conditions used during library screening. Finally, the beads were

transferred to a solution of 10% methanol in water and subjected to UV light (λ = 360 nm) for 45 seconds. The resulting mixture was filtered and subjected to LC-MS/MS analysis.

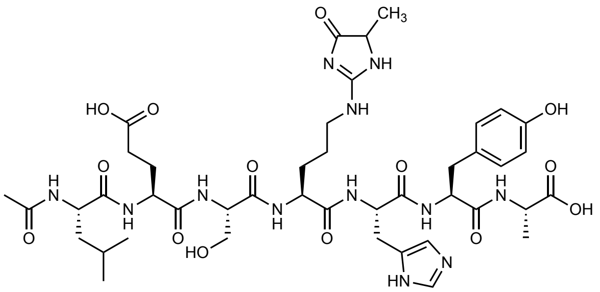

Preparation of MGH-1-Modified Peptide 1 ($1^{MGH\text{-}1}$). For NMR studies, roughly 10 mg of MGH-1 modified peptide **1** was prepared by incubation of peptide **1** (200 µL of 20 mM stock in DMF) with MGO (400 µL of 10 mM stock in water) in phosphate buffered saline (400 µL, pH 12, 100 mM) at a final concentration of 4 mM peptide **1**, 4 mM MGO, 40 mM PBS at pH 12, and 20% DMF in 1 mL total volume. This solution was incubated at 37 °C in a water bath for 3 h. These high pH and higher phosphate concentration conditions were used to promote the formation of MGH-1. The resulting mixture of AGEs, in which MGH-1-modified peptide **1** (peptide $1^{MGH\text{-}1}$) was the predominant product (30-50% yield by LC-MS), was purified by semi-preparative HPLC using a gradient of 10-30% acetonitrile in water over 20 min at 4.0 mL/min. Collected fractions were characterized by MALDI-TOF, pooled and lyophilized. This process was repeated until sufficient quantities of peptide $1^{MGH\text{-}1}$ could be obtained. Stocks of peptide $1^{MGH\text{-}1}$ were assessed for purity by HPLC and LCMS prior to analysis by NMR. For NMR analysis, lyophilized peptide $1^{MGH\text{-}1}$ (ca.10 mg) was dissolved in 550 µL of DMSO-d_6 (roughly 20 mM final concentration) and characterized by NMR. ^{1}H DMSO δ 8.59 (s, 1H), 7.20 (s, 1H), 7.11 (d, *J* = 8.2; 2H), 6.79 (d, *J* = 8.25l; 2H), 4.65 (t, *J* = 7.05; 1H), 4.54 (m, 1H), 4.24-4.33 (m, 3H), 4.33-4.20 (m, 3H), 3.86-3.77 (m, 2H), 3.30 (t, 2H), 3.19 (m, 1H), 3.16-3.00 (m, 3H), 2.90-2.83 (m, 1H), 2.52-2.39 (m, 2H), 2.21-1.88 (m, 3H) 2.03 (s, 3H), 1.78-1.49 (m, 8H), 1.43 (d, *J* = 7.05, 2H), 1.39 (m, 1H), 1.37 (d, *J* = 7.25, 3H), 0.91 (d, *J* = 6.25, 3H), 0.87 (d, *J* = 6.20, 3H).

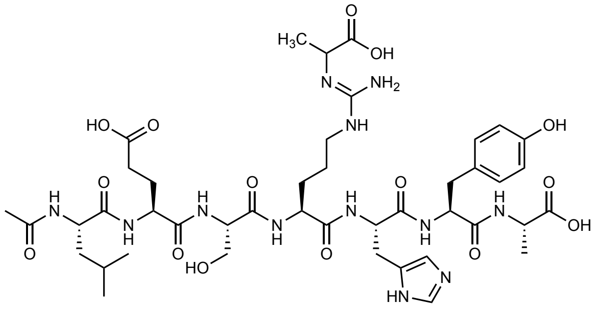

Preparation of CEA-Modified Peptide 1 (1^{CEA}). CEA-modified peptide **1** was prepared by incubating peptide $1^{MGH\text{-}1}$ (100 µL, 20 mM in DMSO) in phosphate buffer (200 µL, pH 7.3, 100 mM) diluted to 1 mL with DI water for final concentrations of 2 mM peptide in 20 mM PBS, 10% DMF. The resulting solution was allowed to incubate at 37 °C for up to 1 week. The resulting mixture of AGEs, in which CEA-modified peptide **1** (peptide 1^{CEA}) was the predominant product, was purified by semi-preparative HPLC using a gradient of 10-30% acetonitrile in water over 20 min at 4.0 mL/min. Collected fractions were characterized by MALDI-TOF, pooled and lyophilized. Stocks of peptide 1^{CEA} were assessed for purity by HPLC and LCMS prior to analysis by NMR. For NMR analysis, lyophilized peptide 1^{CEA} (ca.10 mg) was dissolved in 550 µL of DMSO-d_6 (roughly 20 mM final concentration) and characterized by NMR. ^{1}H DMSO δ 9.22 (s, 1H), 8.48 (s, 1H), 8.16-8.0 (m, 3 H), 7.79 (s, 1H), 7.46 (s, 1H), 7.27 (s, 2H), 7.22 (s, 1H) , 7.12-7.02 (m, 3H), 6.64 (d, 2H), 4.54 (s; 1H), 4.43 (s, 1H), 4.28-4.24 (m, 3H), (t, 2H), 3.10 (m, 1H), 2.96-2.93 (m, 3H), 2.68-2.64 (m, 1H), 2.37 (s,1H) 2.23 (s, 2H), 2.09-1.93 (m, 2H) 1.85 (s, 3H), 1.76-1.59 (m, 5H), 1.48 (s, 2H), 1.43 (m, 1H), 1.34 (d, 3H),1.24 (s, 1H), 01.81 (m, 2H), 0.87 (d, 3H).

NMR Acquisition. ^{1}H and ^{13}C spectra were acquired with a Bruker Advance III (500 MHz, 125 MHz) spectrometer. ^{1}H chemical shifts are reported as in units of parts per million (ppm) relative to DMSO (s 2.50 ppm). Data are reported as follows: chemical shift, multiplicity (s = singlet, d = doublet, t = triplet, q = quartet, m = multiplet), coupling constants (Hz), and integration).

General Protocol for Dilution Experiments. For dilution experiments described in **Main Text Figs. 4** & **5** and **Supplementary Figs. 22** & **23**, peptides (1 mM) were incubated with MGO (1 mM) as described above. After 3 h of incubation at 37 °C, the reactions were diluted 100X (10 µL of reaction mixture diluted in 990 µL buffer) in 20 mM PBS at pH 7.3 at 37 °C for up to 48 additional hours, then subjected to LCMS analysis without any further dilution or purification.

Chemical Derivatization of AGEs using Boronic Acids. Peptides (1 mM final concentration) were treated with MGO (1 mM final concentration) in 20 mM PBS at pH 7.3 with 5% final DMF concentration and heated at 37 °C in a water bath, as previously described. To evaluate boronate ester formation for MGH-DH containing peptides, 25 μL of 200 mM 6-bromo-3-pyridinylboronic acid was added to the reaction solution after 3 h (when MGH-DH predominates) and allowed to incubate for an additional 2 h at 37 °C. For analysis of carboxyethylarginine-containing peptides, 2.5 μL of a 20 mM stock of carboxyethylarginine-modified peptide **1** (peptide $\mathbf{1}^{\mathbf{CEA}}$) in DMSO was diluted into to 37.5 μL water with 10 μL of 10X PBS at pH 7.3 (final concentration 1 mM peptide in 20 mM PBS), and briefly agitated. Subsequently, 25 μL of the peptide $\mathbf{1}^{\mathbf{CEA}}$ solution was combined with 25 μL of 200 mM 6-bromo-3-pyridinylboronic acid and incubated for 2 h (final concentration 100 mM boronic acid) at 37 °C. After incubation, samples were diluted 1:1 with α-cyano-4-hydroxycinnamic acid matrix, spotted on a ground steel plate and subjected to analysis by MALDI.

Chemical Derivatization of AGEs by EDC/HOBt Coupling. Peptides of interest (1 mM final concentration) were incubated with 1-ethyl-3-(3-dimethylaminopropyl)carbodiimide (EDC, 3 mM) and hydroxybenzotriazole (HOBt, 3 mM) in a solution of DMF with 10 mM diisopropyl-ethylamine (DIEA). Following a brief (2-5 min) preincubation of the aforementioned components, ten equivalents (10 mM) of bromo-benzylamine were added to the solution. The resulting mixture was agitated at room temperature for up to 24 hours. To evaluate amide formation at available carboxylate functional groups, 3 μL of each reaction mixture was diluted into 300 μL of pure deionized water (1:100) and subjected to analysis by LC-MS (as previously described) without further purification. The LC-MS mobile phase gradient elution was changed to 5-80% B during a 1.75-16.00 min range to ensure that any hydrophobic reaction products were observed within the chromatographic window. Rather than automated precursor ion selection, targeted ion identification for MS/MS acquisition was used to identify the triply modified derivatization product. After selection in MS, fragmentation was induced using subsequent scans of static collision energies (20, 25, 30, 35V) in each cycle. This strategy allowed for the sampling of multiple fragmentation events that are not captured in when using automated MS/MS acquisition.

Mammalian cell culture. HEK-293T cells (ATCC, CRL-3216) were cultured under standard conditions in Dulbecco's Modified Eagle Medium (DMEM) supplemented with 10% Fetal Bovine Serum (FBS) and 1% Penicillin-Streptomycin (Pen-Strep) at 37 °C at 5% CO_2. Cells were passaged every 2 to 3 days for a maximum of 25-30 passages.

Plasmids for Protein Expression. Plasmids encoding green fluorescent protein (GFP) variants C-terminally fused to the peptide 1 sequence (-LESRHYA, GFP-**1**), the peptide 3 sequence (-LDDREDA, GFP-**3**), or a glycation-inert control sequence (-LESAHYA, GFP-$\mathbf{1}^{\mathbf{Ala}}$) were designed and purchased from GenScript, in a pcDNA_3.1(+) vector, which was selected due to its propensity for uninduced, high expression in mammalian cell lines. These C-terminal peptide sequences were connected to GFP through a linker sequence containing a tobacco etch virus (TEV) protease cleavage site. The expression sequences used to encode the GFP-fusion proteins are shown below. All sequences contain the sequence for the super-folding green-fluorescent protein (b. 1-714), linked via the via the tobacco etch virus protease cleavage sequence (b. 715-735) to the peptide of interest (b. 736-756). The peptide sequence is the only variable region between plasmid sequences.

GFP-**1**:

^{001}ATG AGC AAG GGC GAA GAG CTG TTC ACC GGC GTC GTG CCC ATC CTG GTG GAA CTG^{054}
^{055}GAC GGC GAC GTG AAC GGA CAC AAG TTC AGC GTG CGG GGC GAG GGC GAG GGC GAT^{108}
^{109}GCT ACA AAC GGC AAG CTG ACC CTG AAG TTT ATC TGC ACC ACC GGA AAG CTG CCA^{162}
^{163}GTG CCT TGG CCT ACA CTG GTT ACA ACA CTC ACC TAC GGC GTG CAA TGT TTT AGC^{216}
^{216}AGA TAC CCC GAT CAC ATG AAA AGA CAC GAC TTC TTC AAA AGC GCC ATG CCT GAG^{270}
^{270}GGA TAT GTG CAG GAG AGA ACA ATC AGC TTC AAG GAC GAC GGC ACC TAC AAG ACC^{324}
^{325}AGG GCC GAG GTG AAG TTC GAG GGC GAC ACC CTG GTG AAT AGA ATC GAG CTG AAA^{378}
^{379}GGC ATC GAC TTT AAG GAA GAT GGC AAC ATC CTG GGC CAC AAG CTG GAA TAC AAC^{432}
^{433}TTC AAC AGC CAC AAC GTC TAC ATC ACA GCT GAT AAG CAG AAG AAC GGC ATT AAG^{486}
^{487}GCC AAT TTC AAG ATC CGG CAT AAT GTG GAA GAT GGA TCT GTG CAG CTG GCC GAC^{540}
^{541}CAC TAC CAG CAG AAC ACC CCT ATC GGC GAT GGA CCT GTG CTG CTG CCT GAC AAC^{594}
^{595}CAC TAC CTG TCC ACC CAG AGC GTG CTG AGC AAA GAC CCC AAC GAG AAG CGG GAC^{648}

^{649}CAC ATG GTG CTG CTG GAG TTC GTG ACC GCC GCC GGC ATC ACC CAC GGC ATG GAC^{702}
^{703}GAG CTG TAC AAA GAG AAC CTG TAC TTC CAG TCC CTG GAA TCT AGA CAC TAC GCC^{756}

GFP-**3:**

^{001}ATG AGC AAG GGC GAA GAG CTG TTC ACC GGC GTC GTG CCC ATC CTG GTG GAA CTG^{054}
^{055}GAC GGC GAC GTG AAC GGA CAC AAG TTC AGC GTG CGG GGC GAG GGC GAG GGC GAT^{108}
^{109}GCT ACA AAC GGC AAG CTG ACC CTG AAG TTT ATC TGC ACC ACC GGA AAG CTG CCA^{162}
^{163}GTG CCT TGG CCT ACA CTG GTT ACA ACA CTC ACC TAC GGC GTG CAA TGT TTT AGC^{216}
^{216}AGA TAC CCC GAT CAC ATG AAA AGA CAC GAC TTC TTC AAA AGC GCC ATG CCT GAG^{270}
^{270}GGA TAT GTG CAG GAG AGA ACA ATC AGC TTC AAG GAC GAC GGC ACC TAC AAG ACC^{324}
^{325}AGG GCC GAG GTG AAG TTC GAG GGC GAC ACC CTG GTG AAT AGA ATC GAG CTG AAA^{378}
^{379}GGC ATC GAC TTT AAG GAA GAT GGC AAC ATC CTG GGC CAC AAG CTG GAA TAC AAC^{432}
^{433}TTC AAC AGC CAC AAC GTC TAC ATC ACA GCT GAT AAG CAG AAG AAC GGC ATT AAG^{486}
^{487}GCC AAT TTC AAG ATC CGG CAT AAT GTG GAA GAT GGA TCT GTG CAG CTG GCC GAC^{540}
^{541}CAC TAC CAG CAG AAC ACC CCT ATC GGC GAT GGA CCT GTG CTG CTG CCT GAC AAC^{594}
^{595}CAC TAC CTG TCC ACC CAG AGC GTG CTG AGC AAA GAC CCC AAC GAG AAG CGG GAC^{648}
^{649}CAC ATG GTG CTG CTG GAG TTC GTG ACC GCC GCC GGC ATC ACC CAC GGC ATG GAC^{702}
^{703}GAG CTG TAC AAA GAG AAC CTG TAC TTC CAG TCC CTG GAC GAC AGA GAG GAC GCC^{756}

GFP-**1**$^{\mathbf{Ala}}$:

^{001}ATG AGC AAG GGC GAA GAG CTG TTC ACC GGC GTC GTG CCC ATC CTG GTG GAA CTG^{054}
^{055}GAC GGC GAC GTG AAC GGA CAC AAG TTC AGC GTG CGG GGC GAG GGC GAG GGC GAT^{108}
^{109}GCT ACA AAC GGC AAG CTG ACC CTG AAG TTT ATC TGC ACC ACC GGA AAG CTG CCA^{162}
^{163}GTG CCT TGG CCT ACA CTG GTT ACA ACA CTC ACC TAC GGC GTG CAA TGT TTT AGC^{216}
^{216}AGA TAC CCC GAT CAC ATG AAA AGA CAC GAC TTC TTC AAA AGC GCC ATG CCT GAG^{270}
^{270}GGA TAT GTG CAG GAG AGA ACA ATC AGC TTC AAG GAC GAC GGC ACC TAC AAG ACC^{324}
^{325}AGG GCC GAG GTG AAG TTC GAG GGC GAC ACC CTG GTG AAT AGA ATC GAG CTG AAA^{378}
^{379}GGC ATC GAC TTT AAG GAA GAT GGC AAC ATC CTG GGC CAC AAG CTG GAA TAC AAC^{432}
^{433}TTC AAC AGC CAC AAC GTC TAC ATC ACA GCT GAT AAG CAG AAG AAC GGC ATT AAG^{486}
^{487}GCC AAT TTC AAG ATC CGG CAT AAT GTG GAA GAT GGA TCT GTG CAG CTG GCC GAC^{540}
^{541}CAC TAC CAG CAG AAC ACC CCT ATC GGC GAT GGA CCT GTG CTG CTG CCT GAC AAC^{594}
^{595}CAC TAC CTG TCC ACC CAG AGC GTG CTG AGC AAA GAC CCC AAC GAG AAG CGG GAC^{648}
^{649}CAC ATG GTG CTG CTG GAG TTC GTG ACC GCC GCC GGC ATC ACC CAC GGC ATG GAC^{702}
^{703}GAG CTG TAC AAA GAG AAC CTG TAC TTC CAG TCC CTG GAA TCT GCC CAC TAC GCC^{756}

Cellular MGO Assays. For experiments to evaluate glycation of each GFP variant, roughly 1 million HEK-293T cells were seeded in a 10 cm sterile tissue culture dish and grown to 50-60% confluency (~18-24 hours). The next day, cultures were transfected with 10 μg of the desired plasmid using TransIT-LT1 Transfection Reagent (Mirus Bio, 2 μL/μg plasmid). After transfection, cells were cultured for an additional 24 hours (to >90% confluency) before MGO treatments and subsequent harvesting. In general, cells were treated with MGO diluted into DMEM supplemented with 10% FBS and 1% Pen-Strep, in stocks that were prepared the same day as MGO treatment. MGO stock solutions (100 mM) were prepared in sterile water by the addition of 154 μL of commercially available MGO (40% w/v) to a total volume of 10 mL. The 100 mM MGO stock was further diluted into DMEM until a desired concentration (2.5 or 5 mM final concentration) was reached. Adherent cells on 10 cm sterile culture dishes were treated with 10 mL of this MGO-supplemented DMEM and incubated at 37 °C at 5% CO_2 for up to 3 hours. After MGO treatment, cells were harvested with TrypLE Express (Gibco). Harvested cells were transferred to a 15 mL conical tube, and pelleted at 200 x g. The resulting pellet was washed with 3-5 mL of 20 mM PBS, pH 7.3 and stored on ice until lysis. Cells were lysed on ice in 400-600 μL Tris-Cl buffer (50 mM Tris, 150 mM NaCl, 1 mM EDTA, 1 mM NaF, 1% Triton X-100) at pH 7.5 with a Pierce Protease and Phosphatase Inhibitor tablet (1 tablet/10 mL buffer). Lysates were clarified by centrifugation at 4255 x g for 30 minutes, and total protein quantified by BCA Protein Assay (Pierce) on a Tecan Spark 10M plate reader and analyzed by western blot (10 μg/sample) or used for immunoprecipitation.

Immunoprecipitation Protocol. Cellular lysates (~200-250 μg protein) were diluted to a total volume of 500 μL in Eppendorf tubes containing 50 mM Tris, 150 mM NaCl, 1 mM EDTA, 1 mM NaF, pH 7.5, supplemented with protease and phosphatase inhibitor tablets (Pierce). To this, 25 μL of GFP-Trap® agarose slurry was added and

allowed to incubate for 1-2 hours at 4 °C with end-over-end rotation. Following the incubation period, the agarose resin was pelleted and washed 3X with the same buffer, per the manufacturer's recommendation and transferred to a fritted spin column. For elution using TEV proteolysis, the resin was suspended in 100 µL of 1X TEV Reaction Buffer (New England Biolabs) in a plugged spin column. To this, 100 units of TEV protease (New England Biolabs), were added and incubated at 30 °C, shaking. Following incubation, the supernatant was eluted into a clean Eppendorf tube by brief (30-60 s) centrifugation in a bench-top microcentrifuge. The resulting eluates were collected and subjected to LC-MS analysis with any further purification or dilution. To elute the full length GFP, not only the C-terminal peptide fragment, the protein-bound GFP-Trap® agarose was resuspended in 50 µL of glycine elution buffer provided in the GFP-Trap® enrichment kit (Chromotek). After a 15 min incubation, supernatants were collected by eluting into a clean Eppendorf tube by brief (30-60 s) centrifugation in a bench-top microcentrifuge. Resulting eluates were analyzed by LC-MS (for C-terminal peptide fragments) or by western blot (for full-length protein).

Western Blotting. Western blot analysis was used for cellular lysates and for GFP-Trap® eluates of full-length GFP protein. Lysates with a concentration of 10 µg total protein, or GFP-Trap® elutions were diluted into 6X SDS loading buffer and boiled for 5 minutes. SDS-PAGE was run on pre-cast protein gels (8–16%, mini-PROTEAN® TGX™) in standard Tris/glycine/SDS running buffer at 200 mV for 35 min to resolve protein bands. Following separation by SDS-PAGE, proteins were transferred to PVDF membrane using the iBlot 2 (Invitrogen) dry blotting system. Membranes were subsequently blocked in a buffer containing 20 mM Tris, 150 mM NaCl and 0.1% tween (1X TBST) supplemented with 5% (w/v) skim milk powder. After blocking, primary antibody incubation was achieved overnight at 4 °C per manufacturer's recommendation. Membranes were washed 3X for 5 min each with 5% milk-TBST. Detection was performed by incubating membranes with HRP-linked secondary antibodies (Cell Signaling Technology) in 5% milk-TBST for one hour at room temperature. After secondary antibody incubation, membranes were washed again 3X for 5 min with 1X TBST. Chemiluminescent signal was developed with Clarity Western ECL Substrate (Bio-Rad) and imaged on a Bio-Rad ChemiDoc XRS+.

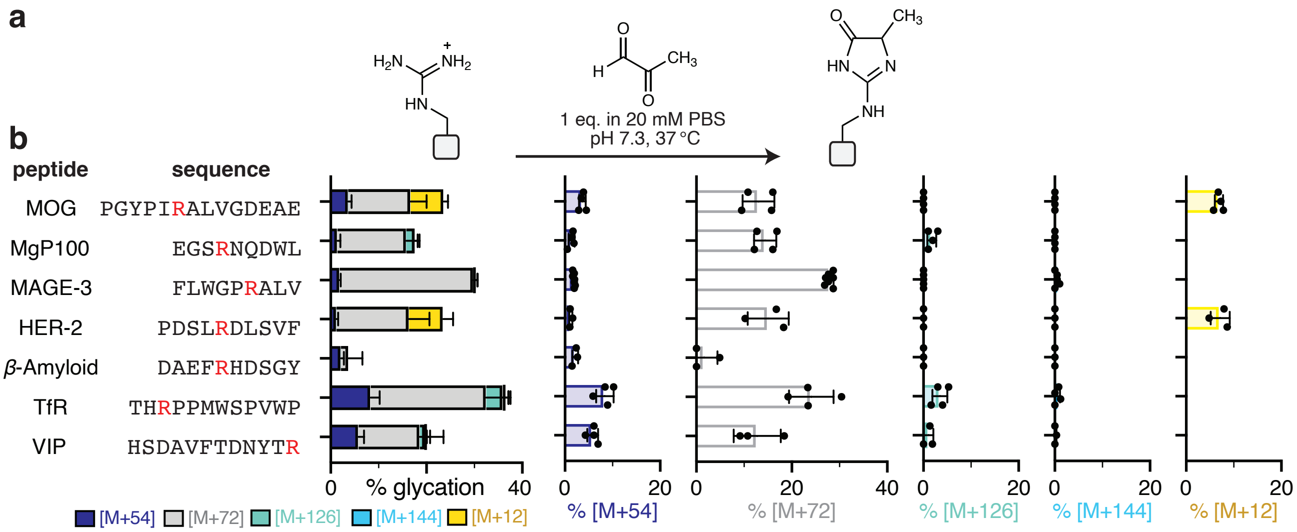

Supplementary Figure 1. Primary Sequence Influences Glycation Outcomes. (a) Reaction scheme used for the glycation of a set of commercially-available peptides. These seven peptides each contain a single arginine, but are varied in their sequence composition and/or length. **(b)** Distribution of AGE adducts observed after treatment with MGO (1 mM, 1 equiv.) for 3 h, as assessed by LC-MS/MS. Stacked bar graphs (left) are plotted as mean ± standard deviation for each mass adduct. Individual data points are shown on the bar graphs provided for each mass adduct (right). These data are derived from independent experiments: MOG (n=4), MgP100 (n=4), MAGE-3 (n=7), HER-2 (n=3), b-Amyloid (n=3), TfR (n=4), VIP (n=4). Legend: blue, [M+54]; gray, [M+72]; green, [M+126]; cyan, [M+144]; yellow, [M+12]. This set of peptides exhibited substantial differences in total glycation, as well as in the diversity of the individual AGE products that formed. Despite the presence of unprotected N-termini, the vast majority of modifications were found at Arg. The major exception was the formation of an [M+12] adduct, which remains structurally undefined, that formed exclusively for peptides with N-terminal Pro residues (see **Supplementary Fig. 2**). These studies support the premise that primary sequence alone, in an otherwise unstructured short peptide, is enough to influence glycation outcomes.

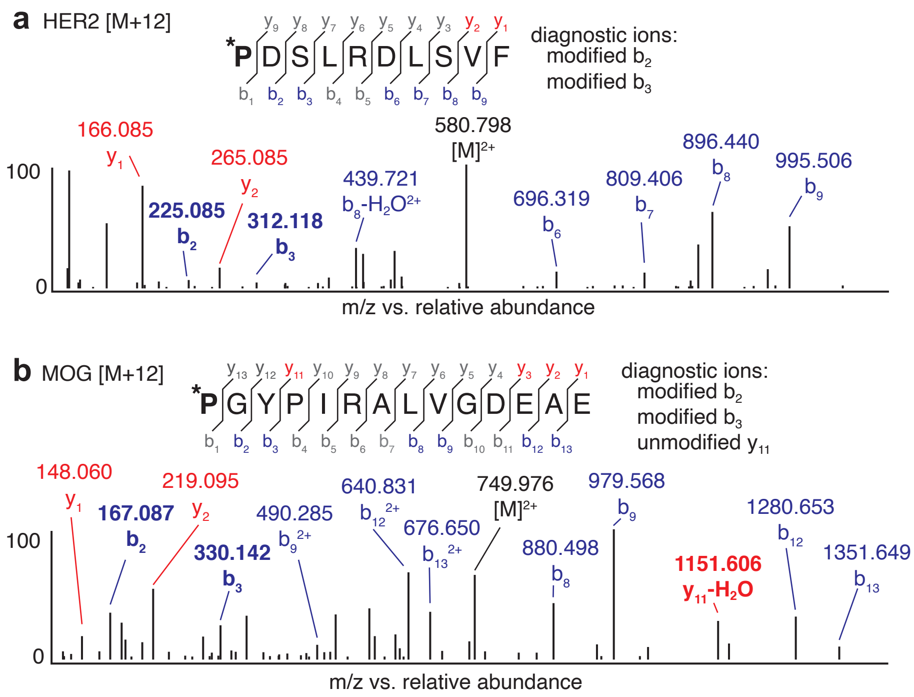

Supplementary Figure 2. An [M+12] AGE is Exclusive to N-terminal Proline. An [M+12] adduct was observed following MGO treatment only for peptides containing an N-terminal proline, including the HER2 peptide and MOG. Using tandem mass spectrometry (LC-MS/MS), it was possible to determine that this adduct results from the modification of N-terminal Pro, not Arg. **(a)** In the HER2 peptide, diagnostic ions such as modified b_2 and b_3 were used to assign the site of modification to the N-terminus. **(b)** For the MOG peptide, diagnostic ions included modified b_2 and b_3, as well as an unmodified y_{11}. This modification did not appear in any other peptide studied in this work.

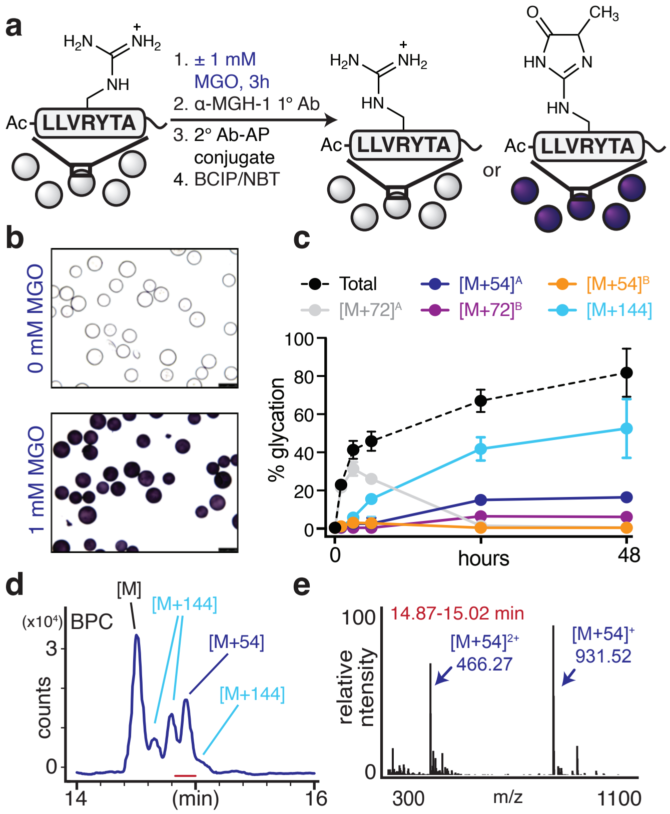

Supplementary Figure 3. Detection of MGH-1-Modified Peptides on Resin. (a) To determine if MGH-1 could be detected on resin-bound peptides, a peptide derived from a control sequence in human serum albumin (-LLVRYTA-) was synthesized on ChemMatrix resin and side chain deprotected (see **Supplementary Materials & Methods** for further detail). After treatment with MGO for 3 h in 20 mM PBS at pH 7.3, and blocking with a 1% solution of bovine serum albumin (BSA) in TBST for 2 h at room temperature, the presence of MGH-1 was detected using commercially-available primary antibodies specific for MGH-1[5] (Cell BioLabs, STA-011). Colorimetric detection was achieved through incubation with secondary antibodies conjugated to alkaline phosphatase and subsequent treatment with BCIP/NBT reagents that form an insoluble blue precipitate upon phosphatase activity. (**b**) After BCIP/NBT treatment, beads were transferred to 25 mm clear plastic dishes and visualized using brightfield microscopy. **(c)** An N-terminally acetylated synthetic peptide matching the control sequence (Ac-LLVRYTA, peptide **4**) was purified and then treated with standard glycation conditions (1 mM MGO, pH 7.3 PBS, 37 °C) for 1 (n=3), 3 (n=5), 6 (n=2), 24 (n=4) or 48 h (n=2), followed by LC-MS. Mean values ± standard deviations are shown. These data were derived from five independent MGO treatment experiments. Legend: black, total glycation; blue, $[M+54]^A$; orange, $[M+54]^B$; gray, $[M+72]^A$; purple, $[M+72]^B$; cyan, [M+144]. Levels of [M+126] were below 5% at all time points tested and are not plotted for the sake of clarity. These studies confirm that an [M+54] mass adduct, which corresponds to the MGH isomers including MGH-1, appears at early time points and persists throughout the entire time course studied for this peptide. However, after early timepoints (1 & 3 h), the single addition adducts become less prominent as double addition adducts rise. We therefore focus on early timepoints as our study has a primary focus on the single addition adduct, MGH-1. We note that this peptide forms two discrete [M+54] adducts with distinct retention times, particularly at early time points (see also **Main Text Fig. 3 and Supplementary Figs. 9, 10 & 23**. **(d)** The representative base peak chromatogram (BPC) from the 24 h timepoint shows the distribution of major AGEs. The [M+144] adduct forms three major isomers. **(e)** A combined mass spectrum for the peak that elutes between 14.87-15.02 min shows the mass adducts corresponding to the [M+54] modification. These studies reveal that peptides that form MGH-1 can be detected on resin using commercially available antibodies.

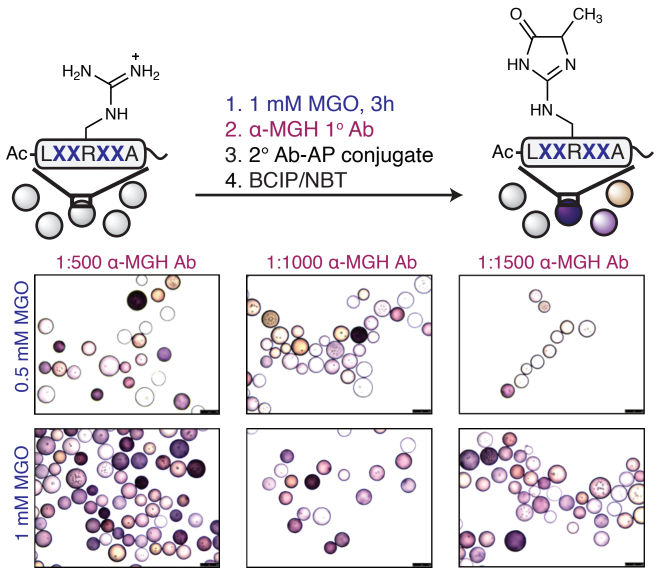

Supplementary Figure 4. Optimization of Library Screening Conditions with α-MGH-1 antibodies. After a one-bead one-compound peptide library was prepared, pilot studies were performed to determine the optimal concentrations of MGO and α-MGH-1 primary antibodies to use for screening. After side chain deprotection and equilibration, beads from a one-bead one-compound peptide library were incubated with either 0.5 mM or 1 mM MGO for 3 h in 20 mM PBS, pH 7.3 at 37 °C. The resulting beads were blocked using a 1% solution of bovine serum albumin (BSA) in TBST for 2 h at room temperature. Next, incubations with different α-MGH-1 primary antibody dilutions (1:500, 1:1000 or 1:1500) were evaluated. These were followed by treatment with secondary antibody and BCIP/NBT color development reagents. Based on these studies, it was determined that treatment with 0.5 mM MGO and incubation with a 1:1000 dilution of the α-MGH-1 antibody maximized both stringency and signal-to-noise.

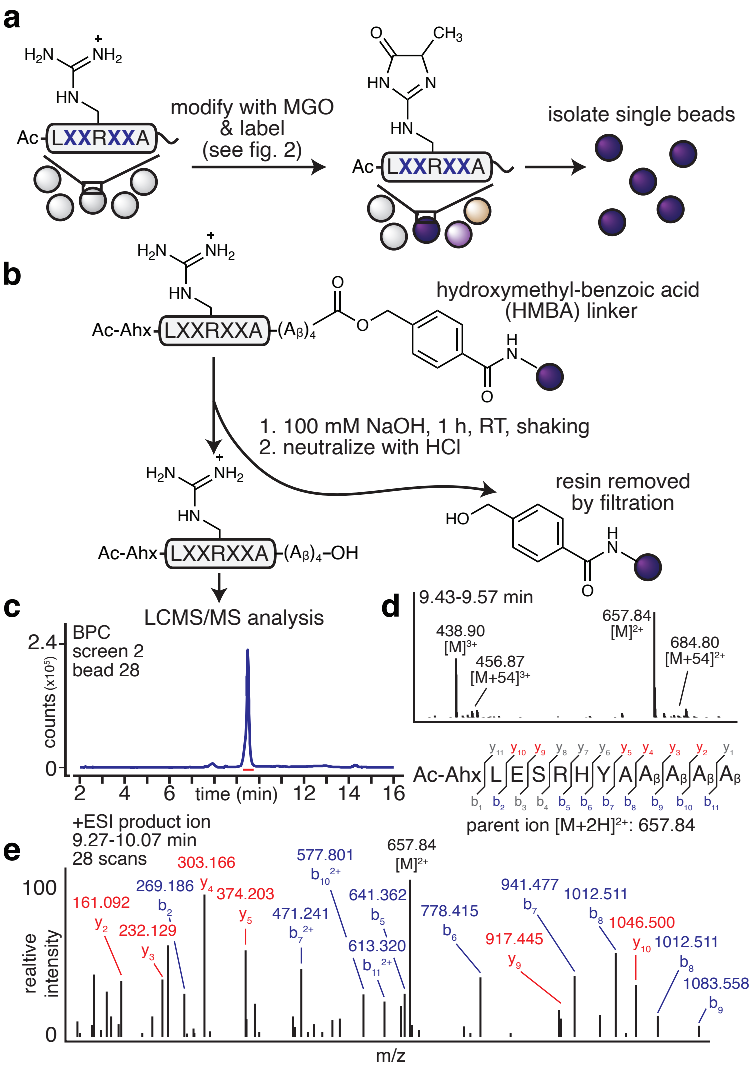

Supplementary Figure 5. Single Bead Sequencing using LC-MS/MS. (a) After treatment with MGO (see **Main Text Fig. 2**), library beads were transferred to a 25 mm clear plastic petri dish and imaged using brightfield microscopy. Beads that had turned dark purple were manually selected for peptide cleavage and analysis by LC-MS/MS. **(b)** Single beads were exposed to 100 mM NaOH for 1 h to cleave the HMBA-ester linkage. After hydroxide base exposure, solutions were neutralized with HCl, filtered, and subjected to LC-MS/MS analysis without further purification. **(c)** A representative LC-MS base peak chromatogram (BPC) for a bead containing the peptide **1** sequence (LESRHYA), collected during library screening. **(d)** The combined mass spectrum for the predominant peptide ion shows a parent ion m/z of 657.84, and low levels of an [M+54] adduct. **(e)** Sequencing by tandem mass spectrometry was accomplished using the *remaining unmodified parent peptide* on each single bead selected. MS/MS data was analyzed using *de novo* sequencing software (PEAKS v 7.5). We did not quantify the distribution of AGEs using this protocol, as strong basic conditions are known to influence AGE outcomes.

Entry	Sequence	Number of Times Selected	Peptide Name
1	L E S R H Y A	6	peptide **1**
2	L E S R Y F A	4	peptide **2f**
3	L E S R Y L A	4	
4	L E S R Y G A	3	
5	L E S R F H A	2	
6	L E S R Y H A	2	
7	L E T R Y S A	2	
8	L E A R H Y A	2	peptide **1c**
9	L E S R Y Y A	2	peptide **2**
10	L H H R E Y A	2	
11	L Q T R Y Q A	2	
12	L A H R Y Q A	1	
13	L D S R Y A A	1	
14	L D G R Y H A	1	
15	L E G R Y A A	1	
16	L E S R Y A A	1	
17	L E T R D F A	1	
18	L E Q R H F A	1	
19	L E T R H F A	1	
20	L E G R Y F A	1	
21	L E G R Y G A	1	
22	L E T R Y G A	1	
23	L E F R H H A	1	
24	L E Y R S H A	1	
25	L E N R Y H A	1	
26	L E H R Y Q A	1	
27	L E N R Y S A	1	
28	L E S R Y S A	1	
29	L E G R H Y A	1	
30	L E Q R H Y A	1	
31	L E T R H Y A	1	
32	L E A R N Y A	1	
33	L E G R N Y A	1	
34	L E H R S Y A	1	
35	L G A R Y Q A	1	
36	L G A R H Y A	1	
37	L H N R Y E A	1	
38	L H G R D F A	1	
39	L H G R E Q A	1	
40	L H G R Y Q A	1	
41	L H G R Y T A	1	
42	L H G R Y V A	1	
43	L H T R E Y A	1	
44	L H T R N Y A	1	
45	L N H R D Y A	1	
46	L N S R H Y A	1	
47	L Q Q R S Y A	1	
48	L S Y R H Q A	1	
49	L S S R Y Q A	1	
50	L S T R Y Q A	1	
51	L T H R Y Q A	1	
52	L Y T R D H A	1	
53	L Y G R E H A	1	
54	L Y D R N H A	1	
55	L Y G R H Q A	1	
	Total:	75	

Supplementary Table 1. Hit Sequences Identified. Sequences that promoted MGH-1 formation were identified using colorimetric detection following incubation with a commercially-available primary antibody specific for MGH-1. Using this approach, 75 individual "hit" beads were selected, cleaved and sequenced using LC-MS/MS and PEAKS *de novo* sequencing software. Of the 75 beads selected, 55 unique peptide sequences were identified.

a

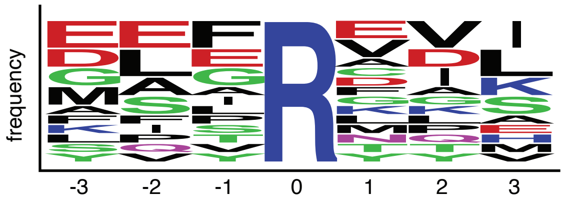

b

DST**R**TQI	LAS**R**LYA	DQA**R**ELI	GLF**R**VAS	EAG**R**EVV
KVV**R**FDK	AEE**R**YPI	GII**R**NVL	MEE**R**KIK	EEF**R**ADH
SSL**R**DIL	ELY**R**GGL	MPF**R**VTE	YLP**R**MKM	FFG**R**CVS

Supplementary Figure 6. Arg-Containing Sequences from Ovalbumin Do Not Match Consensus Motif. The commercially available polyclonal α-MGH-1 antibody (Cell BioLabs, STA-011) was raised against heterogeneously glycated ovalbumin.[5] To confirm that our library selection strategy did not introduce a preference for these potential epitopes, we evaluated all arginine-containing sequences from ovalbumin **(a)** as a frequency logo or **(b)** individually. We found that none of these sequences, or even similar sequences, were selected in the peptide library (see **Supplementary Table 1**), confirming that α-MGH-1 antibodies are indeed specific for MGH-1 modified Arg side chains.

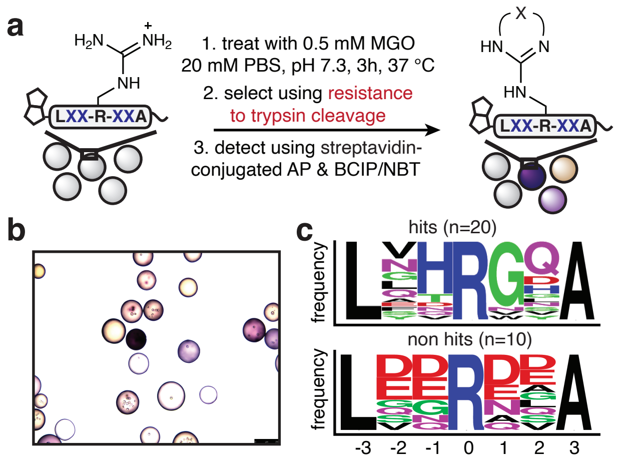

Supplementary Figure 7. Library Screening Using Impaired Proteolysis by Trypsin. To confirm that we selected sequences that promote MGH-1 formation when using α-MGH-1 primary antibodies to screen our library, we also developed an alternative approach that could distinguish hit beads based on impaired proteolysis by trypsin. This alternative screening strategy was able to select sequences that promote high levels of glycation overall, but that is independent of the formation of specific AGEs, such as MGH-1. (**a**) To prepare this library, randomized sequences were capped with an N-terminal biotin in place of an acetyl group (see **Supplementary Methods**). After the same MGO treatment steps (0.5 mM MGO), beads were subjected to proteolytic cleavage by trypsin. Trypsin cleavage is impaired when Arg becomes glycated. Thus, the N-terminal biotin was removed only for *unmodified* sequences. The remaining glycated sequences were selected following incubation with a streptavidin-alkaline phosphatase conjugate and visualization with colorimetric BCIP/NBT reagents. **(b)** Representative image demonstrating that after this treatment, many beads display peptides that remained unmodified and were therefore colorless after exposure to trypsin and colorimetric reagents. However, a few beads were highly glycated, as indicated by the dark purple color (center). These dark purple beads were manually selected, cleaved, and sequenced using LC-MS/MS and PEAKS *de novo* sequencing software. **(c)** Using this approach, just 20 individual "hit" beads were identified. The consensus motif that was obtained using this strategy (LVHRGQA) is distinct from the one derived from the α-MGH-1 screening approach (LESRYYA). Sequences that lead to the lowest levels of overall glycation were identified by choosing beads that were virtually colorless (see **(b)**). The consensus motif obtained for these 10 "non-hits" was significantly different from those obtained for "hit" beads from either screening approach, and indicated that an abundance of negative charge is detrimental to glycation (see also **Supplementary Table 2**), corroborating our previous findings.[2] We note that the consensus motif that was obtained using impaired proteolysis by trypsin is different from the one obtained when using α-MGH-1 antibodies. These results are consistent with a model in which the "trypsin" approach selects for any modified glycation adduct, which could include more than ten known chemical structures that can form just from the reaction between MGO and Arg.[7–9] It is most likely that the trypsin approach screens for the first steps of the glycation reaction that occur rapidly, such as the formation of MGH-DH (see **Main Text Fig. 4**), whereas the α-MGH-1 screening approach provides information about features that promote the (presumably rate-determining) elimination step that converts MGH-DH to MGH-1 (see also **Main Text Figs. 4** & **6**).

Entry	Sequence	Number of Times Selected	Peptide Name
1	**L D D R E D A**	**1**	peptide **3**
2	**L D G R D L A**	**1**	
3	**L D Q R A D A**	**1**	
4	**L E E R N Q A**	**1**	
5	**L E G R D V A**	**1**	
6	**L E N R D S A**	**1**	
7	**L G D R Q G A**	**1**	
8	**L Q D R D A A**	**1**	
9	**L S E R N E A**	**1**	
10	**L V E R E E A**	**1**	
	Total:	10	

Supplementary Table 2. Non-Glycated Sequences Possess Dense Negative Charge. Sequences that are resistant to modification by MGO were identified using trypsin proteolysis (See **Supplementary Fig. 7**). Library beads that remain unmodified upon treatment with MGO are able to be cleaved by trypsin to remove the N-terminally linked biotin motif. Sequences were identified by selecting beads with no color development upon treatment with alkaline phosphatase-conjugated streptavidin and color development by BCIP/NBT reagents. Because the trypsin cleavage was not 100% efficient, it was possible to sequence peptides selected from colorless beads based on the low levels (~10%) of full-length peptide that remained following trypsin exposure. Ten beads were selected, cleaved and sequenced using LC-MS/MS and PEAKS *de novo* sequencing software. In all cases, there is a substantial over-representation of acidic residues, Asp and Glu. Entry 1 (-LDDREDA-) is a hit that matches well with the consensus motif (see **Supplementary Fig. 7**) and was therefore chosen as a representative non-glycated control sequence for subsequent peptide studies.

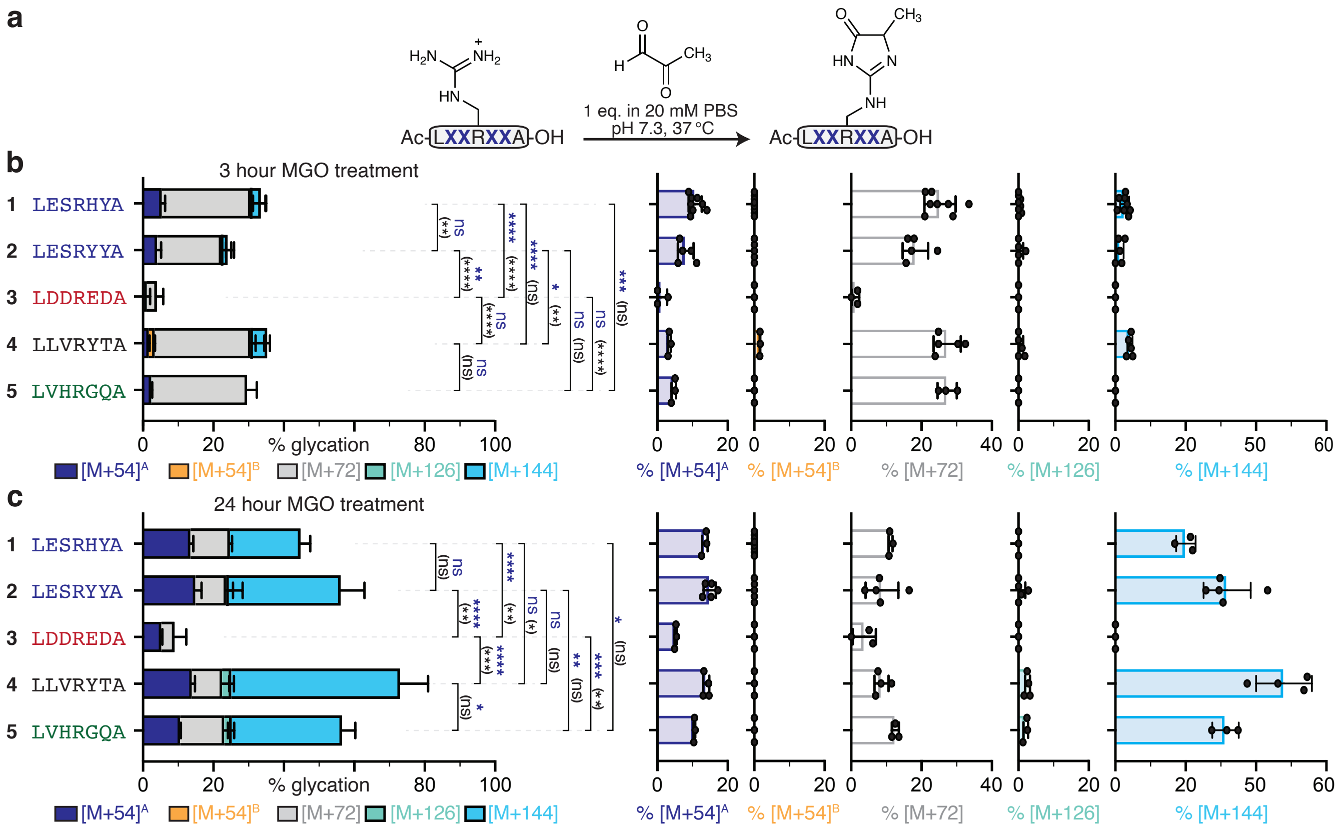

Supplementary Figure 8: Validation of Library-Derived "Hit" and "Non-Hit" Peptides. (a) Purified synthetic peptides that were N-terminally acetylated were allowed to react with equimolar MGO (1 mM) in 20 mM PBS pH 7.3 for **(b)** 3 h or **(c)** 24 h at 37 °C and were then analyzed by LC-MS/MS. Stacked bar graphs (left) are plotted as mean ± standard deviation for each mass adduct. Individual data points are plotted for each mass adduct (right). Legend: blue, $[M+54]^A$; orange, $[M+54]^B$; gray, [M+72]; green, [M+126]; cyan, [M+144]. **(b)** Distribution of AGE adducts observed for peptides **1-5** (Ac-LESRHYA (**1**), Ac-LESRYYA (**2**), Ac-LDDREDA (**3**), Ac-LLVRYTA (**4**), and Ac-LVHRGQA (**5**)) Data were derived from independent experiments: peptide **1** (n=8), peptide **2** (n=5), peptide **3** (n=3), peptide **4** (n=5), peptide **5** (n=3). Importantly, the hit sequence obtained when screening with α-MGH-1 antibodies (peptide **1**) yields significantly more [M+54] than both a control sequence from HSA (peptide **4**) and the hit sequence obtained when screening using resistance to trypsin proteolysis (peptide **5**), in as early as 3 hours of MGO treatment. The "non-hit" sequence (peptide **3**) yields significantly less glycation and less [M+54] than any other peptide tested. **(c)** Distribution of AGE adducts observed for peptides **1-5** (Ac-LESRHYA (**1**), Ac-LESRYYA (**2**), Ac-LDDREDA (**3**), Ac-LLVRYTA (**4**), and Ac-LVHRGQA (**5**) after 24 hours of MGO treatment. Data were derived from independent experiments: peptide **1** (n=3), peptide **2** (n=5), peptide **3** (n=3), peptide **4** (n=4), peptide **5** (n=3). There are still significant differences in [M+54] and total glycation levels for peptides **1, 2, 4 & 5** compared to the negative control sequence, peptide **3**. However, differences in the amount of [M+54] are overshadowed by large increases in the double addition adduct, [M+144], which likely corresponds to isomers of tetrahydropyridine (see **Fig. 1**), or other structurally uncharacterized MGO double addition adducts. A non-directional (two-tailed), one-way ANOVA using Tukey's multiple comparison test was used to compare the mean levels of [M+54] adducts (blue) or total glycation (black) for all peptides. $p<0.05$(*), $p<0.01$(**), $p<0.001$(***), $p<0.0001$(****). Additional statistical analysis is available in the Supplementary Data 1 file.

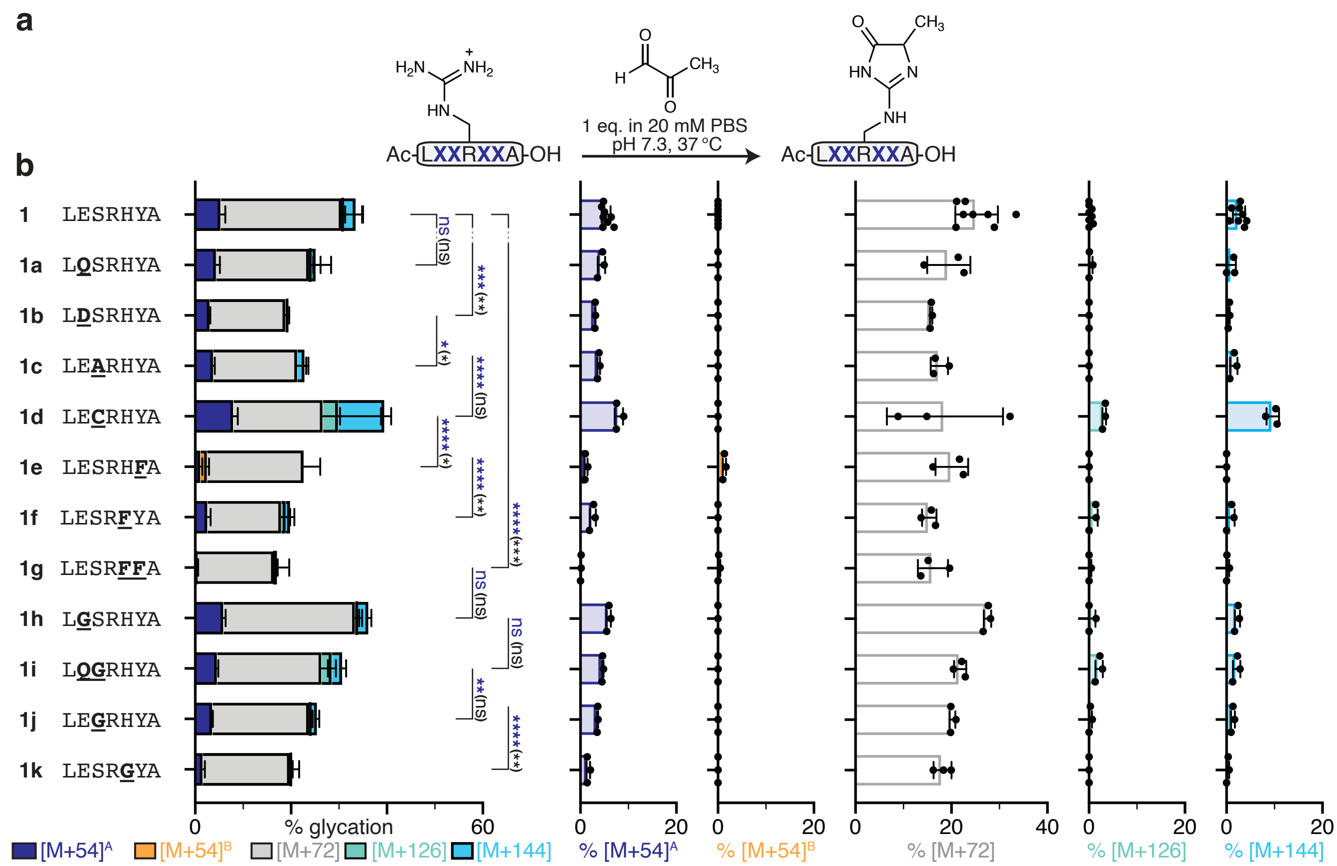

Supplementary Figure 9. Point Variants of Peptide 1. (a) Scheme depicting the glycation of peptide **1** and its variants by MGO. **(b)** Levels of glycation and distributions of AGE adducts were quantified by LC-MS, based on at least three replicate experiments. Stacked bar graphs (left) are plotted as mean ± standard deviation for each mass adduct. Individual data points are plotted for each mass adduct (right). Legend: blue, [M+54]^A; orange, [M+54]^B; gray, [M+72]; green, [M+126]; cyan, [M+144]. Data are derived from independent experiments: peptide **1** (n=8), peptide **1a** (n=3), peptide **1b** (n=3), peptide **1c** (n=3), peptide **1d** (n=3), peptide **1e** (n=3), peptide **1f** (n=3), peptide **1g** (n=3), peptide **1h** (n=3), peptide **1i** (n=3), peptide **1j** (n=3), peptide **1k** (n=3). Peptides **1a-g** are shown here and in **Main Text Fig. 3**. Replacing Glu in the -2 position with Gln (**1a**) led to a subtle decrease in total glycation that is not statistically significant. There were also no statistically significant changes in the [M+54] adduct. This can be attributed to a conservative substitution that retains critical polar contacts. In contrast, replacement with Asp (**1b**) led to a substantial decrease in both MGH-1 and total glycation levels, suggesting that the specific placement of the Glu side chain is critical. The substitution of Ser in the -1 position with Ala (**1c**) also led to a moderate decrease in total glycation levels and a small, but in this case statistically significant, decrease, in [M+54] levels. Conversely, the Cys substitution (**1d**) led to notable increases in [M+54] and the [M+126] and [M+144] double addition adducts. Notably, we did not observe any formation of a recently identified intramolecular glycation crosslink, MICA.[10] When Tyr in the +2 position was substituted with Phe (**1e**), a substantial decrease in [M+54] levels was observed. This was also the case for the substitution of His in the +1 position with Phe (**1f**). Levels of total glycation and, in particular, [M+54] were reduced even farther by double substitution of His and Tyr with Phe (**1g**). Additional point variants include the Glu to Gly mutation (**1h**) as well as the double mutation of Glu to Gln and Ser to Gly (**1i**), which both exhibit minimal changes. A substitution of Ser to Gly (**1j**) yields a decrease in [M+54] levels but no statistically significant change in overall glycation compared to peptide **1**. The substitution of His to Gly (**1k**) results in significantly less total glycation and decreased levels of [M+54]. A non-directional (two-tailed), ordinary one-way ANOVA using Dunnett's multiple comparison test to compare each mean to the mean for peptide **1**, was used to determine if each variant yielded significantly different amounts of [M+54] (blue) or total glycation (black). $p<0.05$(*), $p<0.01$(**), $p<0.001$(***), $p<0.0001$(****). Additional statistical analysis is available in the Supplementary Data 1 file.

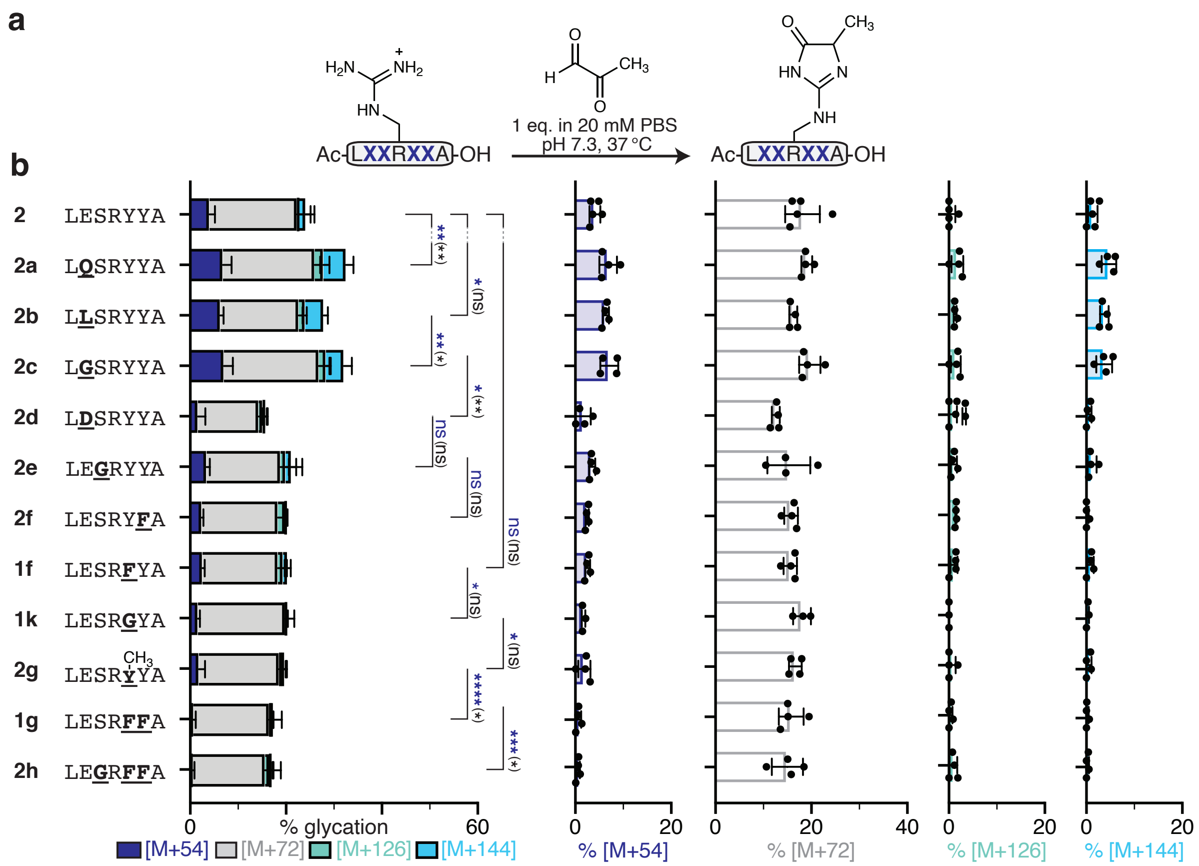

Supplementary Figure 10. Point Variants of Peptide 2. A similar series of variants were prepared and evaluated for peptide **2**, which represents the consensus motif obtained from the library. **(a)** Scheme depicting the glycation of peptide **2** and its variants by MGO. **(b)** Levels of glycation and distributions of AGE adducts were quantified by LC-MS, based on at least three replicate experiments. Stacked bar graphs (left) are plotted as mean ± standard deviation for each mass adduct. Individual data points are plotted for each mass adduct (right). Legend: blue, [M+54]; gray, [M+72]; green, [M+126]; cyan, [M+144]. Data are derived from independent experiments: peptide **2** (n=5), peptide **2a** (n=4), peptide **2b** (n=4), peptide **2c** (n=4), peptide **2d** (n=4), peptide **2e** (n=4), peptide **2f** (n=4), peptide **1f** (n=4), peptide **1k** (n=3), peptide **2g** (n=4), peptide **1g** (n=4), peptide **2h** (n=4). A mutation of Glu to Gln (**2a**), Leu (**2b**) or Gly (**2c**) all yield statistically significant increases in [M+54], and in some cases total glycation, which can be attributed to the loss of negative charge that has been shown to be detrimental to glycation (see also **Supplementary Table 2** & **Supplementary Figs. 7, 8** & **9**). In a similar pattern to peptide **1**, the mutation of Glu to Asp (**2d**) led to a substantial decrease in overall glycation as well as in the formation of [M+54]. Single mutations of Ser to Gly (**2e**) or Tyr to Phe (peptides **2f** & **1f**) led to no significant changes in glycation. However, substitution of the Tyr in the +1 position with Gly (**1k**) does significantly lower [M+54]. The same effect is achieved through O-methylation this same Tyr (**2g**). Removal of both Tyr (**1g**) produces the most drastic decrease in [M+54], further substantiating the claim that tyrosine is critical for the formation of [M+54], particularly MGH-1. A third substitution of Ser with Gly (**2h**) did not lead to any further reduction in glycation. A non-directional (two-tailed) ordinary one-way ANOVA using Dunnett's multiple comparison test, to compare each mean to the mean for peptide **2**, was used to determine if variants yield significantly different amounts of [M+54] (blue) or total glycation (black). p<0.05(*), p<0.01(**), p<0.001(***), p<0.0001(****); (****); n≥3 for each peptide tested. Additional statistical analysis is available in the Supplementary Data 1 file.

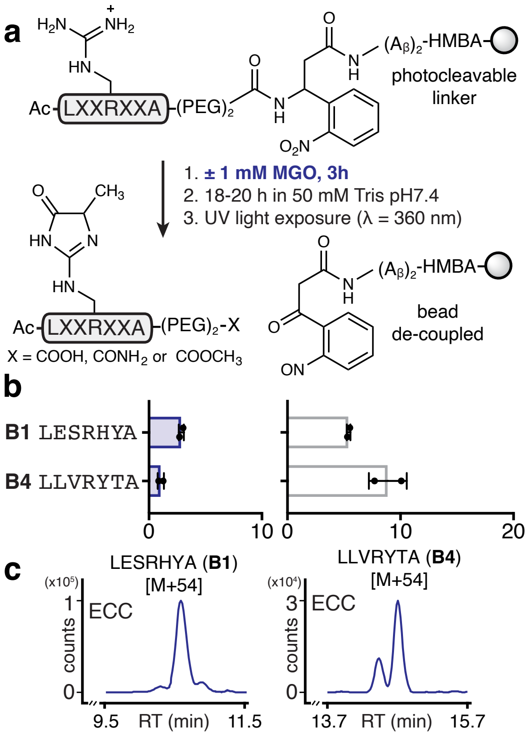

Supplementary Figure 11. Analysis of Glycation on Resin. Typical cleavage protocols used to liberate modified peptides from ChemMatrix resin require the use of strong base, which can influence the resulting distribution of AGEs. **(a)** To quantify the extent of glycation on-resin, it was necessary to develop an alternative cleavage protocol. To do so, the most abundant hit sequence (LESRHYA, peptide **B1**), and a control sequence derived from HSA (LLVRYTA, peptide **B4**), were both synthesized on ChemMatrix resin using a photocleavable linker, ANP (see **Methods**). After MGO exposure and wash steps that mimicked the conditions used during library screening, glycated sequences were cleaved from the bead using high intensity UV light. Due to the nature of ANP linker cleavage, three ions corresponding to the acid, amide or methyl ester C-terminus were observed for each modified sequence. **(b)** Quantification of the extent and distribution of glycation for both peptides provided a clear rationale for why LESRHYA was selected from the library, but LLVRYTA was not: Although levels of total glycation were somewhat greater for **B4** than for **B1**, the latter showed more [M+54] adduct, which is detected by the α-MGH-1 antibodies. Data shown in (b) are derived from independent experiments (n=2). **(c)** Additionally, extracted compound chromatograms (ECCs) for the observed [M+54] adducts show that peptide **B4** led to multiple [M+54] isomers whereas peptide **B1** yielded just one (see also **Main Text Fig. 3**). The representative ECCs shown are for the [M+54] adduct to amidated peptide obtained after photocleavage. Similar results were observed for methyl ester and acid products.

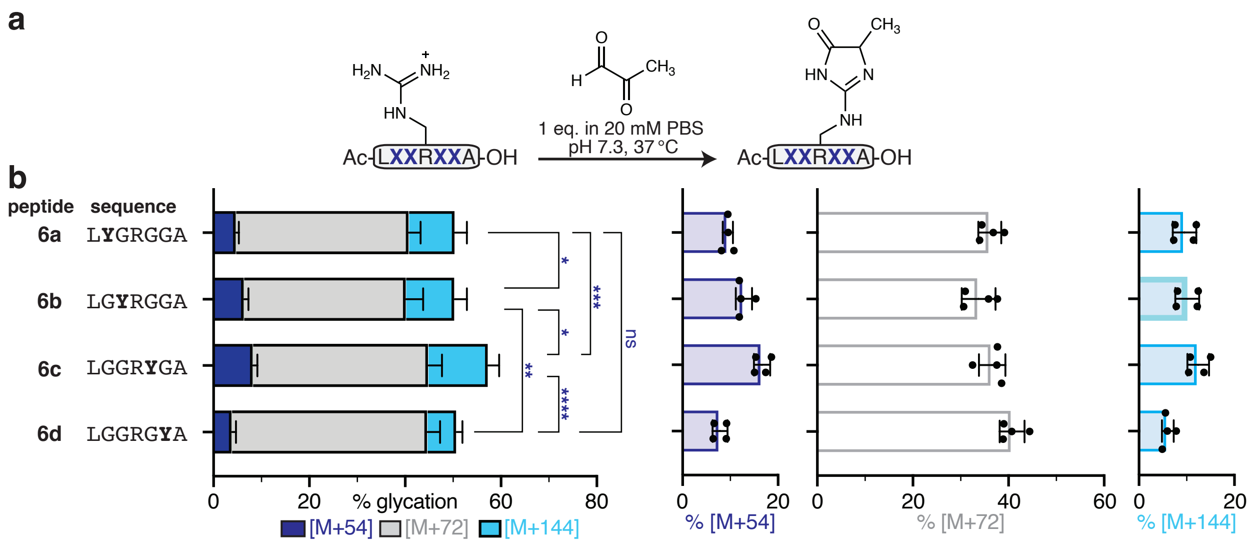

Supplementary Figure 12. Mapping the Positional Contribution of Tyr. Having demonstrated the importance of Tyr in peptides **1** & **2**, we sought to evaluate how the placement of Tyr relative to the central Arg influences glycation outcomes. To do so, we synthesized a series of peptides with Tyr in each of the variable positions, and Gly in all other positions that were randomized (peptides **6a-d**). **(a)** Scheme depicting the glycation of these peptides by MGO. **(b)** As in **Main Text Fig. 3**, levels of glycation were quantified by LC-MS (n=4). Stacked bar graphs (left) are plotted as mean ± standard deviation for each mass adduct. Individual data points are plotted for each mass adduct (right). Legend: blue, [M+54]; gray, [M+72]; cyan, [M+144]. Data are derived from four independent replicate experiments. In these studies, we found that the overall extent and distribution of AGEs was fairly similar for each peptide. In all cases, a single [M+54] isomer was observed. However, when Tyr was placed immediately C-terminal to Arg (peptide **6c**), it yielded significantly more [M+54] than any other peptide in the series. Peptides in this series exhibit greater amounts of the single and double addition adducts [M+72] and [M+144]. Additionally, these peptides all produced more overall glycation (>50%) than any other peptide series evaluated in this study, including peptide **1**. The interplay between total glycation and formation of specific adducts is quite complicated. The overall increase in glycation in these peptides can mostly be attributed to the increase in additions that yield [M+72] and [M+144] adducts. We have observed that residue identity surrounding a central arginine is critical for influencing the mechanistic steps that result in AGE formation. For instance, the dense negative charge in peptide **3** likely disrupts initial MGO addition, as evidenced by the drastic decrease in [M+72] adduct at all timepoints (see **Fig. 2** & **Supplementary Fig. 8**). Furthermore, the position of tyrosine, relative to arginine and other amino acids in the sequence, is critical in favoring [M+54] adducts. This suggests that the additional functionalities in the peptide **1** scaffold serve to favor MGH-1 formation, rather than promote overall glycation (see also **Supplementary Fig. 13**). A non-directional (two-sided) ordinary one-way ANOVA using Tukey's multiple comparison test was used to compare the mean yields of [M+54] (blue) or total glycation (black) for all peptides. $p<0.05$(*), $p<0.01$(**), $p<0.001$(***), $p<0.0001$(****). Additional statistical analysis is available in the Supplementary Data 1 file.

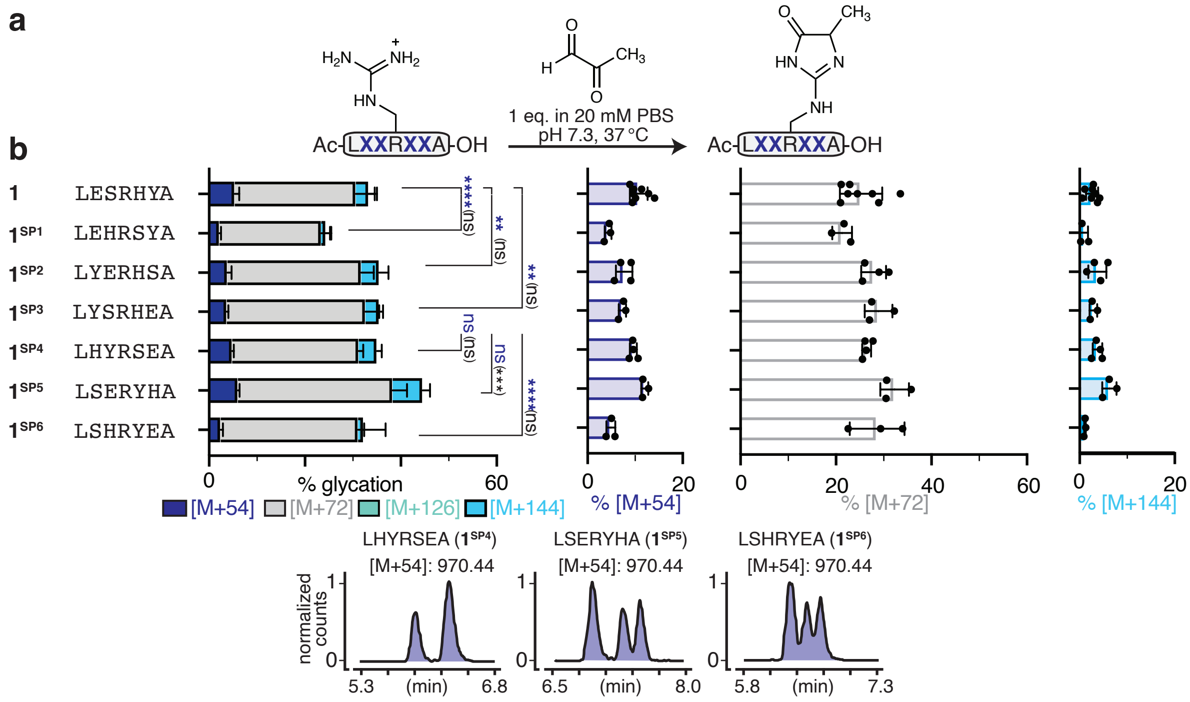

Supplementary Figure 13. Scrambled Peptide 1 Variants Suggest Cooperative Interactions Influence Glycation Outcomes. In a further attempt to understand how the position of the residues within peptide **1** influence glycation, we synthesized a series of scrambled peptide **1** variants (peptides $\mathbf{1^{SP1}}$-$\mathbf{1^{SP6}}$) that each possess the same amino acids, but in a different sequence. **(a)** Scheme depicting the glycation of these peptides by MGO. **(b)** Levels of glycation were quantified by LC-MS. Stacked bar graphs (left) are plotted as mean ± standard deviation for each mass adduct. Individual data points are plotted for each mass adduct (right). Legend: blue, [M+54]; gray, [M+72]; green, [M+126]; cyan, [M+144]. Data are derived from independent experiments: LESRHYA(n=8), LEHRSYA (n=3), LYERHSA (n=4), LYSRHEA (n=3), LHYRSEA (n=4), LSERYHA (n=3), LSHRYEA (n=3). We found that the overall level of glycation as well as the distribution of AGEs could be influenced by scrambled peptide **1** variants. Importantly, we found that several scrambled variants (peptide $\mathbf{1^{SP4}}$, $\mathbf{1^{SP5}}$, and $\mathbf{1^{SP6}}$) produced multiple [M+54] isomers, whereas peptide **1** produces just one. Interestingly, all peptides that yielded multiple isomers of [M+54] contain a tyrosine directly adjacent to arginine in sequence. Though we have confirmed that tyrosine's activity as a base is critical, the relative position of other residues likely contribute to formation of the necessary intermediates that contribute to the overall mechanism for MGH-1 formation (see **Main Text Fig. 6**). This result strongly suggests that Tyr alone is not sufficient to produce the specific glycation outcome, and instead Tyr works cooperatively with other residues in the peptide **1** scaffold to produce a single MGH isomer. A non-directional (two-sided) ordinary one-way ANOVA using Dunnett's multiple comparison test to compare each mean to the mean for peptide **1** was used to determine if variants yield significantly different amounts of [M+54] (blue) or total glycation (black). p<0.05(*), p<0.01(**), p<0.001(***), p<0.0001(****). Additional statistical analysis is available in the Supplementary Data 1 file.

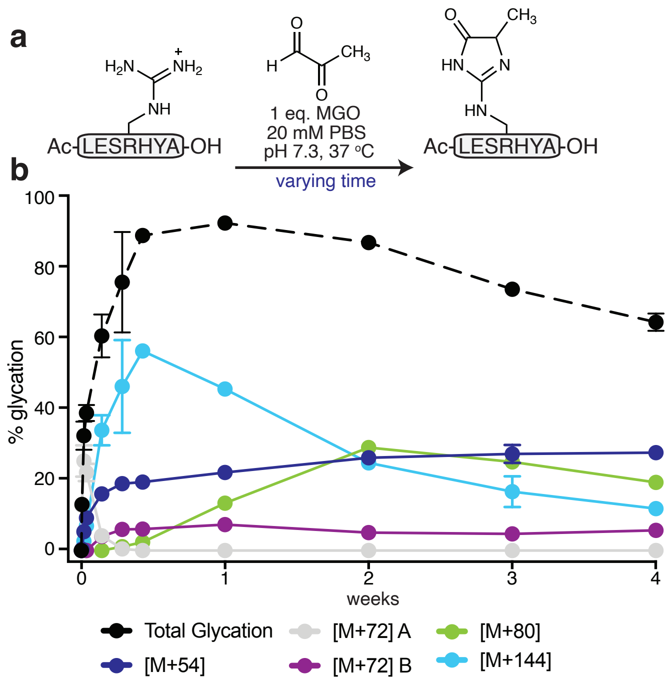

Supplementary Figure 14. Extended Glycation Reactions Reveal MGH-1 as a Predominant AGE. (a) Scheme depicting the glycation of peptides **1** with MGO for up to 4 weeks of incubation. **(b)** Distributions of AGE adducts were quantified after 1, 3, 6, and 24 hours of incubation, then daily for up to 3 days and weekly up to four weeks. Data are plotted as are mean values ± standard deviation, derived from independent experiments. For each timepoint, the number of independent measurements of all adducts at each timepoint were as follows: 1 h (n=3); 3 h (n=8); 6 h (n=2); 24 h (n=3); 48 h (n=3); 72 h (n=3); 1 wk (n=3); 2 wk (n=3); 3 wk (n=3) 4 wk (n=3). Legend: black, total glycation; blue, [M+54]; gray, $[M+72]^A$; purple, $[M+72]^B$; lime, [M+80]; cyan, [M+144]. We found that total glycation levels rose for up to one week, but then began to fall, presumably as less stable AGEs begin to decay. In particular, the [M+144] adduct (cyan), which likely corresponds to the MGO double addition THP, peaks after 3 days, and then decays steadily for the remaining time in the 4 weeks studied. The loss of [M+144] corresponds to a nearly identical gain in [M+80], likely APY (see **Main Text Fig. 1**) between 3 and 14 days, perhaps suggesting a mechanistic link between these two AGEs. However, in the final 2 weeks studied, levels of the [M+144] and [M+80] adducts decay. In contrast, MGH-1 levels climb rapidly in the first 3 days to 19.3 ± 0.3% and continue to climb slowly to 26.2 ± 1.5% by two weeks and then remain consistent for the remainder of the time studied. These studies support previous proposals that MGH-1 is a stable AGE.

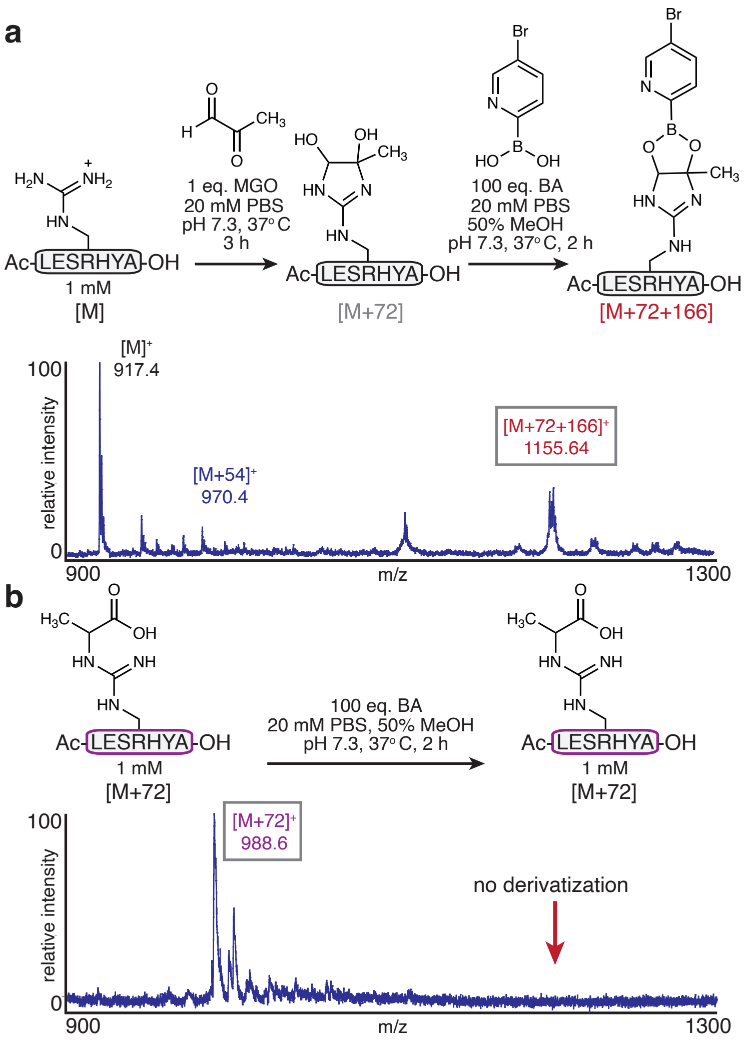

Supplementary Figure 15. Chemical Derivatization of Peptide 1 [M+72] Adducts. MGH-DH is a single addition of MGO to arginine. The structure of MGH-DH includes a vicinal diol that comes from the formation of two hemiaminals between the aldehyde and ketone of MGO and the guanidino group on Arg. We hypothesized that this bis-hemiaminal could be derivatized using boronic acids, which have been reported to derivatize vicinal diols on sugars.[11] **(a)** Scheme for the chemical derivatization of the peptide **1** $[M+72]^A$ adduct (RT = 9.35 min) using boronic acids. We found that the resulting boronic esters were not stable to LC-MS conditions, and thus these reactions were evaluated using MALDI. Treatment of this first $[M+72]^A$ adduct, which dominates early timepoints, with a brominated boronic acid resulted in formation of a new peak corresponding to the boronic ester, which also possessed the characteristic isotope pattern expected for bromine. **(b)** The second $[M+72]^B$ adduct (RT =9.86 mins), which becomes the major product only after 48 h of dilution (see **Main Text Fig. 4**) is unable to be derivatized by boronic acids using the identical protocol, suggesting it to be consistent with formation of carboxyethylarginine (CEA) (see also **Supplementary Figs. 17 & 18**).

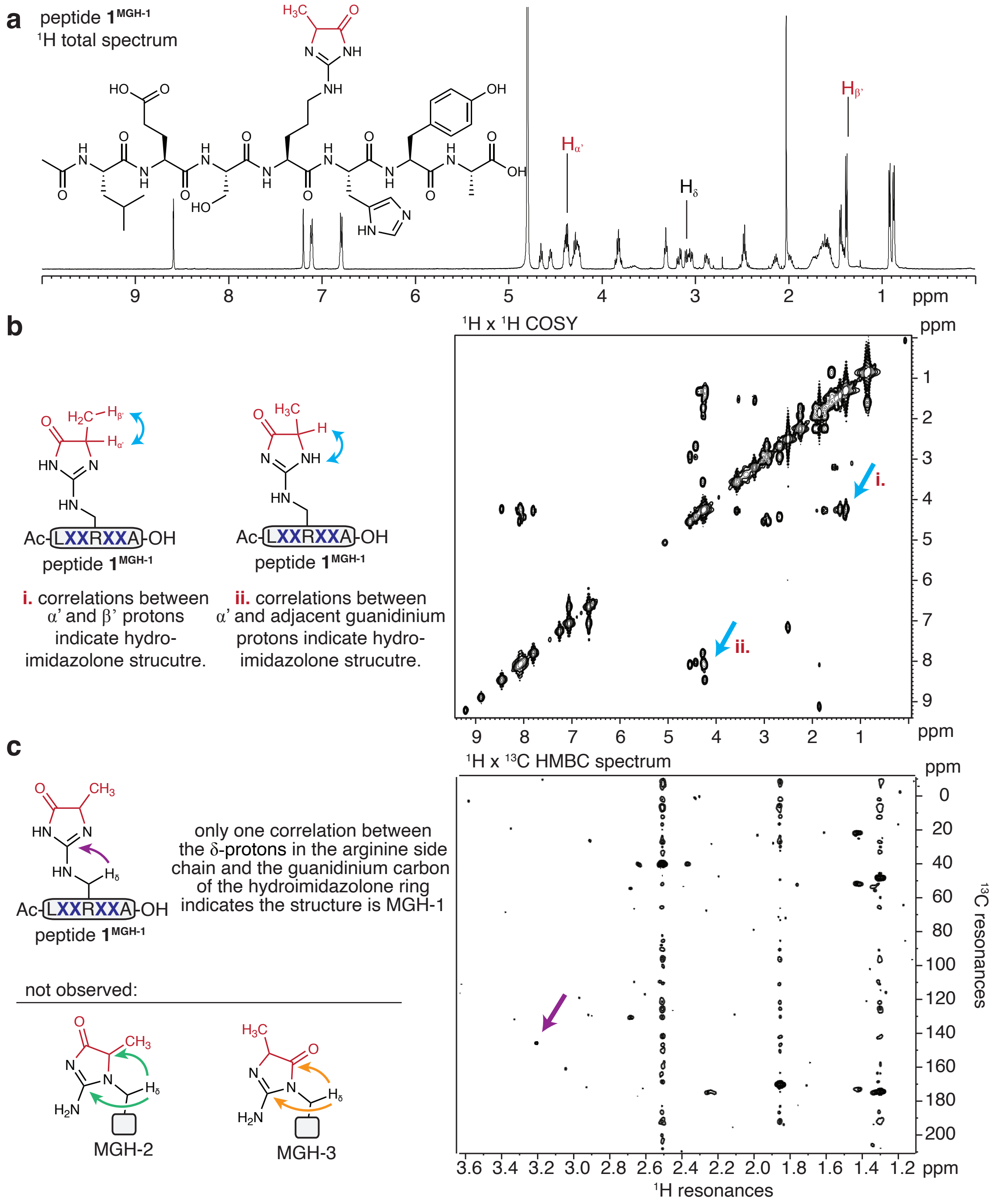

Supplementary Figure 16. Characterization of Peptide 1^{MGH-1} by NMR. Sufficient quantities of peptide **1**$^{MGH-1}$ were prepared to allow for structural studies by NMR. **(a)** The ^{1}H spectrum is shown with key resonances highlighted, including the MGH-1 α-proton (**H**$_{α'}$; δ = 4.40-4.33, m, 3H due to overlap with other α-protons) and methyl-protons (**H**$_{β'}$; δ = 1.36, d, *J* = 7.25, 3H) as well as the Arg side chain δ-protons (**H**$_{δ}$; δ = 3.15, d, 2H). **(b)** Using COSY, (i) it was possible to observe the correlation between the MGH-1 α-proton (**H**$_{α'}$) and methyl-protons (**H**$_{β'}$), which confirms the structure as a hydroimidazolone ring. (ii) In addition, correlations between the MGH-α-proton and the guanidinium protons also confirm the hydroimidazolone structure. **(c)** To differentiate between each of the three possible hydroimidazolone isomers, we relied on three-bond heteronuclear multiple bond correlations (HMBC) between the **H**$_{δ}$ protons of the Arg side chain and carbons in the hydroimidazolone ring, shown with purple arrows. Structures of the three possible MGH isomers are shown to the left. MGH-1 would have only one HMBC correlation, whereas MGH-2 and MGH-3 would each have two. As seen in the HMBC spectra (right), only one HMBC correlation is observed for **H**$_{δ}$, indicating the modification is indeed MGH-1.

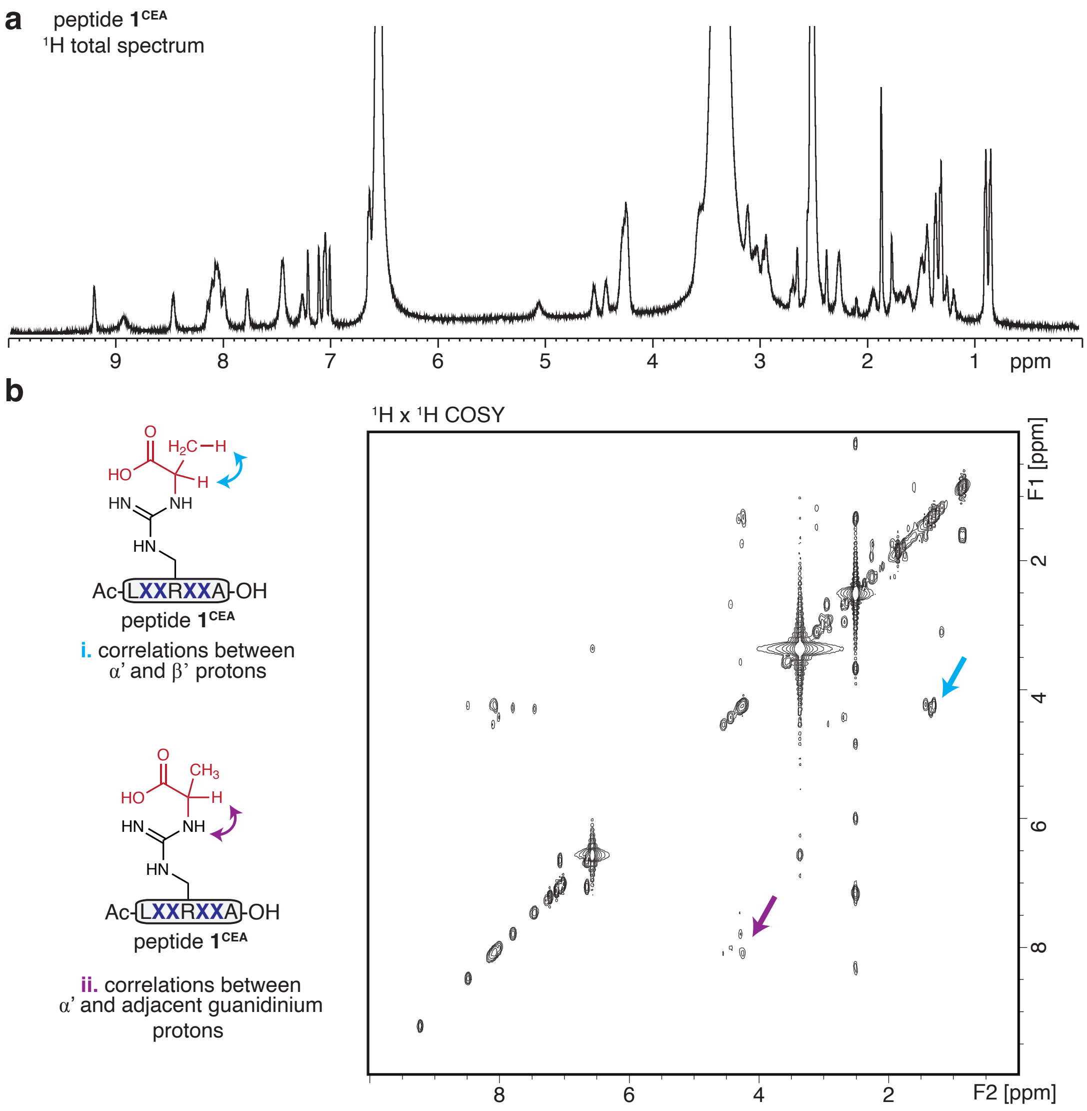

Supplementary Figure 17. Characterization of Peptide 1^{CEA} by NMR. Sufficient quantities of peptide **1^{CEA}** were prepared to allow for structural studies by NMR. **(a)** The ^{1}H spectrum is shown. **(b)** Key resonances between the α-protons (Hα'; δ = 4.28-4.24, m, 3H - due to overlap with other peptide alpha-protons) and methyl-protons (H_{β}'; δ = 1.34, d, 3H) are shown in the COSY, indicated with a cyan arrow. Another diagnostic resonance, the correlation of guanidinium protons (δ = 8.16-8.00, m, 3H) are shown with a purple arrow. These correlations distinguish this adduct as CEA and exclude possibility of this structure being the other prominent [M+72] adduct, MGH-DH, which would lack these correlations.

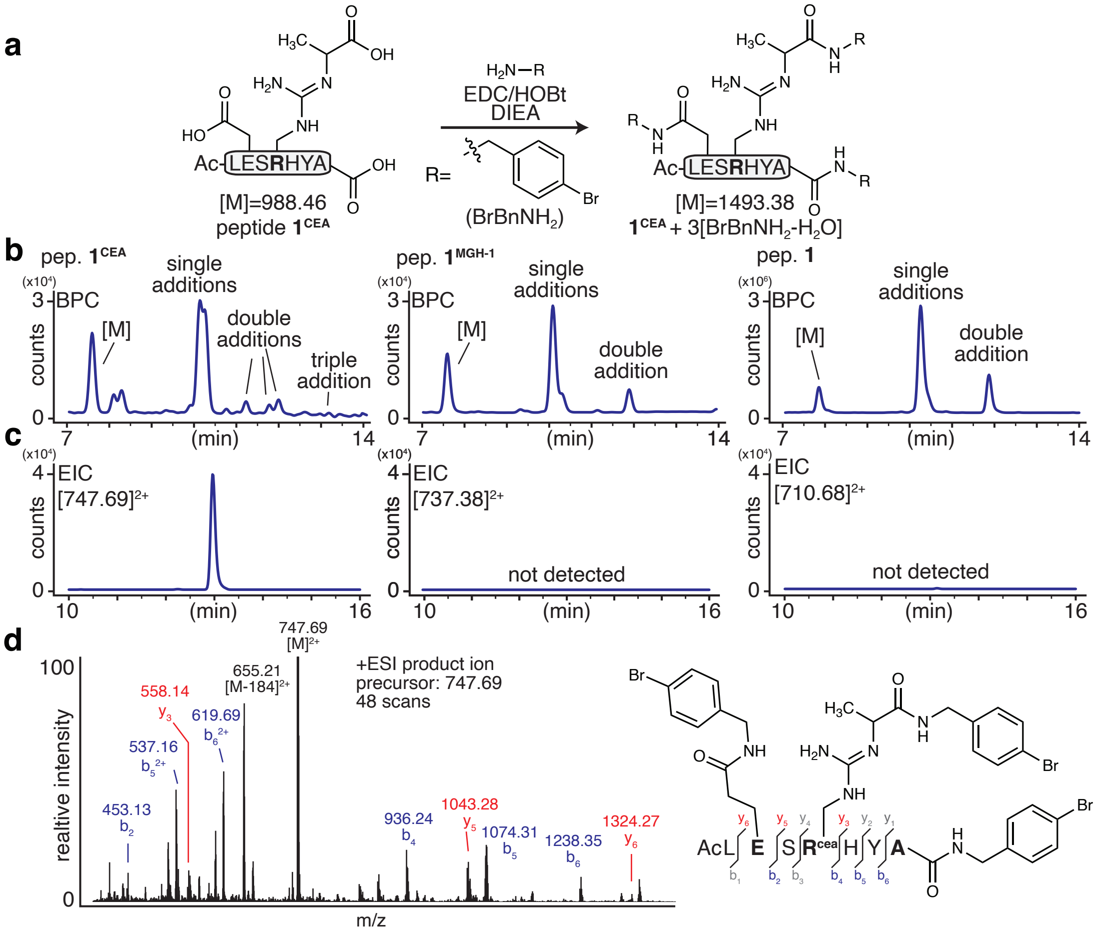

Supplementary Figure 18. Chemical Derivatization of Peptide 1^{CEA}. To chemically characterize peptide $\mathbf{1}^{CEA}$, we treated peptides with amide-bond forming reagents. **(a)** Purified $\mathbf{1}^{CEA}$**,** $\mathbf{1}^{MGH\text{-}1}$ or **1** (1 mM) was incubated in DMF with 3 equiv. (3 mM) EDC, and 3 equiv. (3 mM) HOBt, in a solution of 10 mM DIEA in DMF. After a brief (2-5 min) incubation with these activating reagents, 10 equiv. of bromo-benzyl amine (10 mM) were added and the resulting reaction mixture was agitated at room temperature for up to 24 hours. To evaluate the amount of acyl-substitution, the mixture was diluted 1:100 into pure deionized water and analyzed by LC-MS. **(b)** BPCs for each peptide $\mathbf{1}^{CEA}$, $\mathbf{1}^{MGH\text{-}1}$, and **1** show the extent of acyl-substitution. **(c)** Extracted Ion Chromatograms (EICs) for triply modified peptide for each parent peptide are shown. Notably, all peptides largely undergo just single additions of bromo-benzylamine. Importantly, peptide $\mathbf{1}^{CEA}$, the only peptide to contain three carboxylate functional groups, is the only peptide to undergo substitution three times (as determined by mass). **(d)** The resulting parent ion for the triple modification (747.69) was selected for targeted MS/MS ionization. The resulting product ion spectrum contains ions that are consistent with the three modifications at glutamate (**E**), CEA-modified arginine (**R**^{CEA}) and the C-terminus (**A**). The two peaks in **(b)** between 8-8.5 min for $\mathbf{1}^{CEA}$ are column impurities, not related to the reaction.

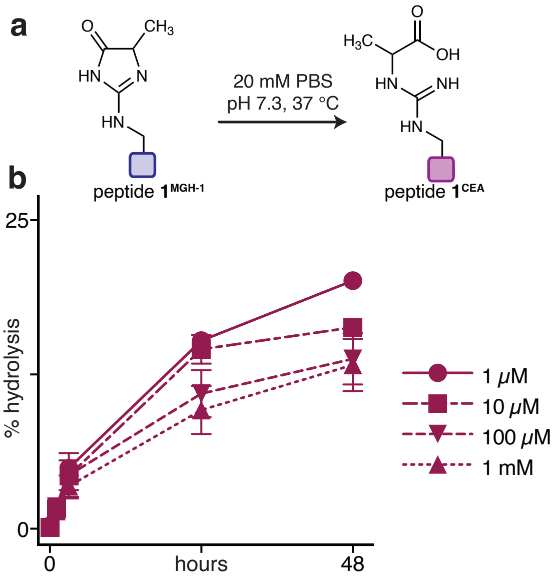

Supplementary Figure 19. MGH-1 is Directly Converted to CEA. **(a)** To determine if MGH-1 can form CEA, purified samples of peptide **1**$^{\mathbf{MGH\text{-}1}}$ were prepared and diluted into 20 mM PBS, pH 7.3, at 1 μM, 10 μM, 100 μM, or 1 mM final concentrations. **(b)** These samples were incubated at 37 °C for up to 48 hours, and the extent of MGH-1 hydrolysis to yield CEA was determined as a "% hydrolysis" based on the observed [M+18] mass shift and distinct retention time for the peptide **1** $[M+72]^B$ adduct. Data are plotted as mean values ± standard deviation, derived from independent experiments. 1 mM: 0 h (n=4), 1 h (n=3), 3 h (n=3), 24 h (n=3), 48 h (n=3); 10 mM: 0 h (n=5), 1 h (n=5), 3 h (n=5), 24 h (n=5), 48 h (n=4); 100 mM: 0 h (n=5), 1 h (n=5), 3 h (n=4), 24 h (n=3), 48 h (n=5); 1 mM: 0 h (n=5), 1 h (n=5), 3 h (n=3), 24 h (n=5), 48 h (n=5). No other adducts were formed during this incubation period: after 48 h, only peptide **1**$^{\mathbf{MGH\text{-}1}}$ and peptide **1**$^{\mathbf{CEA}}$ are present, as seen for the BPCs provided in **Main Text Fig. 5**.

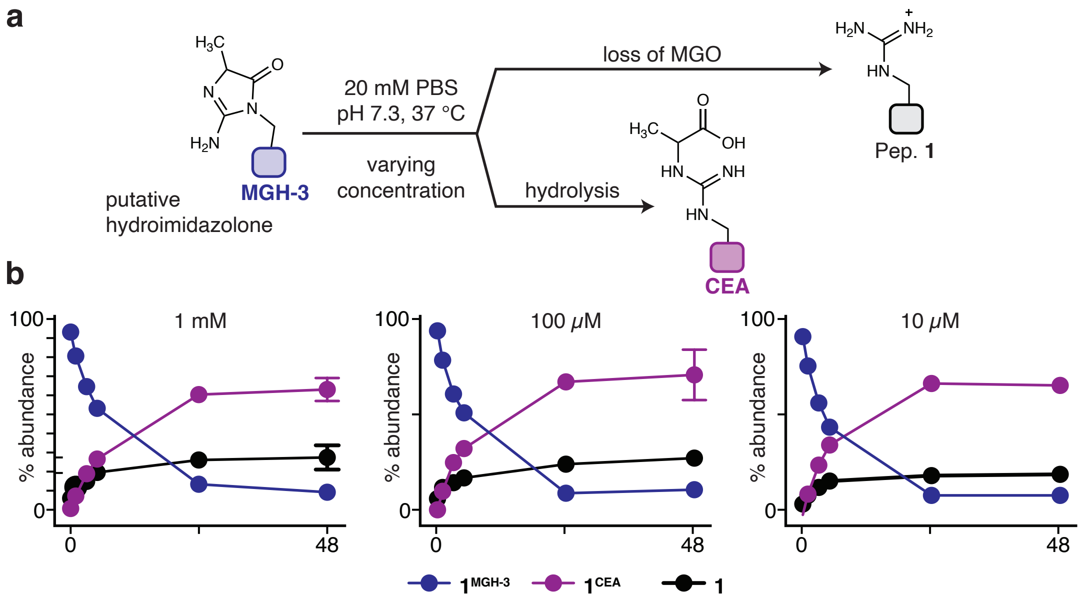

Supplementary Figure 20. A Putative Peptide 1 MGH-3 Adduct Rapidly Produces CEA. (a) Using alkaline conditions (pH 12), it was possible to identify a second MGH isomer that accumulates in a small yield (< 5%) at short times, and likely corresponds to MGH-3. We purified this putative MGH-3 peptide **1** adduct and evaluated its ability to form CEA by resuspending it at 1 mM, 100 µM, or 10 µM concentrations in PBS at pH 7.3. **(b)** After incubation at 37 °C, we determined the resulting distribution of products using LC-MS. Data are plotted as mean values ± standard deviation, derived from independent hydrolysis experiments. 10 mM: 0 h (n=3), 1 h (n=3), 3 h (n=3), 5 h (n=2), 24 h (n=2), 48 h (n=2); 100 mM: 0 h (n=3), 1 h (n=3), 3 h (n=3), 5 h (n=3), 24 h (n=3), 48 h (n=3); 1 mM: 0 h (n=3), 1 h (n=3), 3 h (n=3), 5 h (n=3), 24 h (n=3), 48 h (n=3). We found that the putative MGH-3 isomer undergoes rapid hydrolysis to form CEA (~70% at all concentrations) and also undergoes AGE removal to regenerate unmodified peptide **1** (~25%). After 48 h of incubation, the putative MGH-3 adduct was nearly quantitatively consumed. Compared to MGH-1 hydrolysis (see **Supplementary Fig. 19**), there appears to be less concentration dependence in these studies, although there are small differences in the rate of appearance of CEA at early timepoints. However, all concentrations tested led to similar levels product distributions (predominantly CEA and unmodified peptide **1**) by the end of the 48 h incubation period. Because MGH-3 is known to be less stable than MGH-1 and more prone to hydrolysis, these observations are consistent with formation of a peptide **1-MGH-3** adduct. We note that the observed profile of CEA formation for peptide **1** upon dilution (see **Main Text Figs. 4** & **5**), tracks closely with that which is observed for peptide $\mathbf{1}^{\mathbf{MGH\text{-}1}}$, not the putative MGH-3 adduct. If MGH-3 were to form, we would expect to see greater levels of CEA formation along with greater levels of unmodified peptide **1** from which AGEs were removed. Levels of glycation for peptide 1 only drop very slightly upon dilution (see **Main Text Figs. 4** & **5** and **Supplementary Figs. 22-23**). Thus, we conclude that the relevant intermediate in our study is indeed MGH-1. Together, our results are most consistent with a model in which MGH-1 is a direct precursor to CEA (see also **Main Text Fig. 4, Main Text Fig. 5** and **Supplementary Fig. 19**).

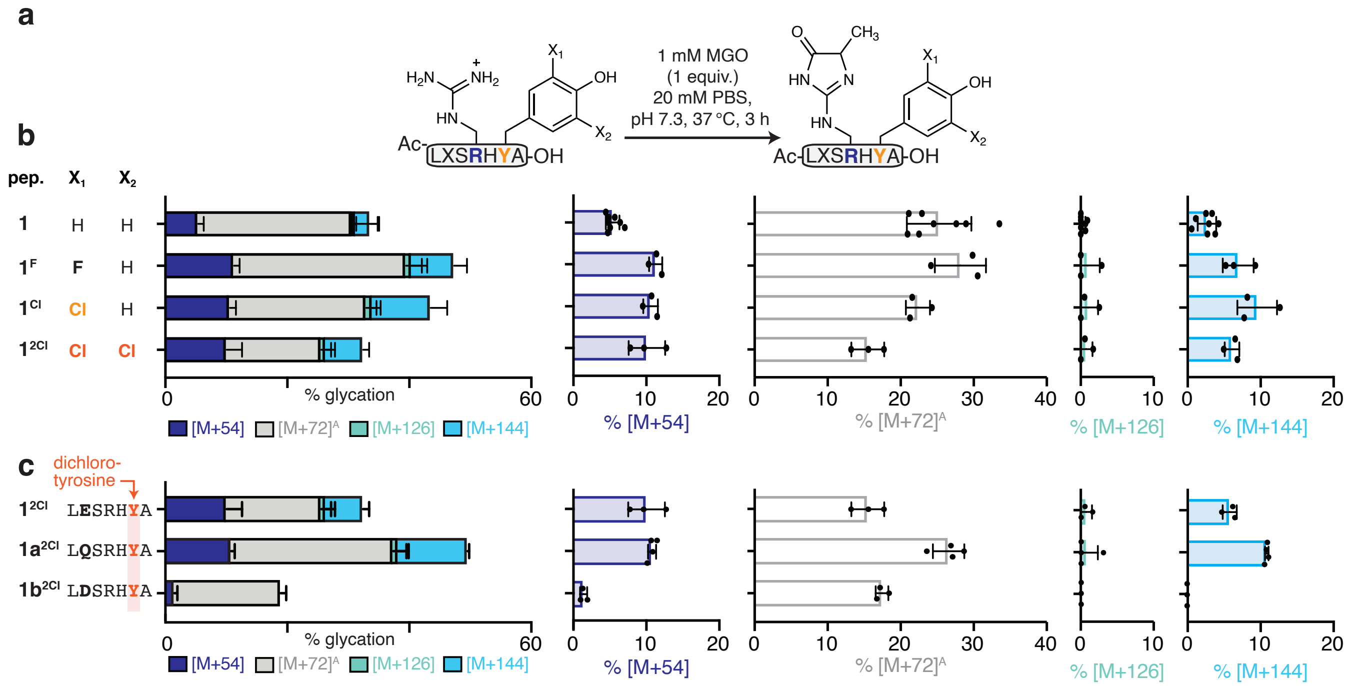

Supplementary Figure 21. Companion to Main Text Figure 6. The same data that appear in **Main Text Figure 6a and 6d** are replotted here to show individually plotted data values. **(a)** Scheme depicting the glycation of these peptides by MGO. **(b)** Distribution of AGEs observed for peptides **1**, **1**^{F}, **1**^{Cl} and **1**^{2Cl} after 3 h of MGO treatment, which also appear in **Main Text Fig. 6a**. **(c)** Distribution of AGEs observed for peptides **1**^{2Cl}, **1a**^{2Cl}, and **1b**^{2Cl} after 3 h of MGO treatment, which also appear in **Main Text Fig. 6d**. Data plotted in **(b)** and **(c)** are mean values ± standard deviation derived from independent MGO treatment experiments. peptide **1** (n=8); **1**^{F} (n=3); **1**^{Cl} (n=3); **1**^{2Cl} (n=3), **1a**^{2Cl} (n=3); **1b**^{2Cl} (n=3). Legend: blue, [M+54]; gray, [M+72]^{A}; green, [M+126]; cyan, [M+144]. Additional statistical analysis is available in the Supplementary Data 1 file.

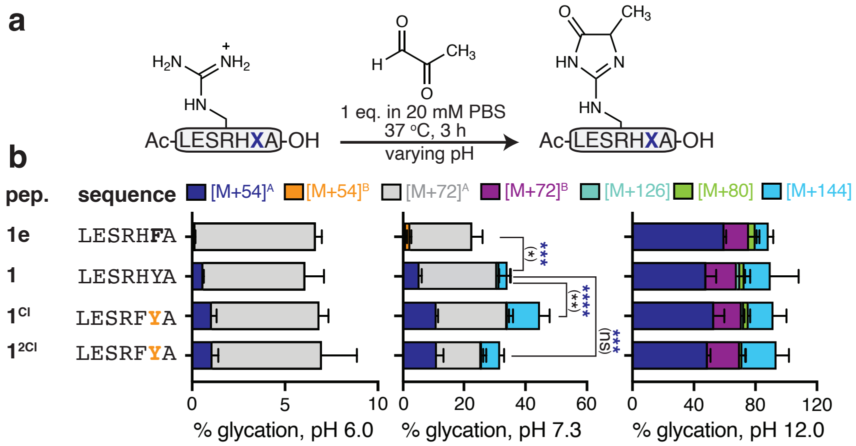

Supplementary Figure 22. Evaluating Glycation at Different pH. To investigate the hypothesis that the Tyr phenoxide acts as a general base, facilitating the conversion of MGH-DH to MGH-1, we prepared chlorinated variants of peptide **1** (peptides $\mathbf{1}^{Cl}$ and $\mathbf{1}^{2Cl}$). **(a)** Scheme depicting the glycation of this series of peptides with MGO. These experiments were completed using our standard MGO reaction conditions, with the exception of the phosphate buffer pH, which was adjusted to either pH 6.0 or pH 12. At pH 6.0, all phenolic groups are expected to be predominantly protonated and uncharged. At pH 12.0, all phenolic groups are expected to be deprotonated and negatively charged. However, at pH 7.3, Tyr would be expected to be protonated and uncharged for peptides **1** and $\mathbf{1}^{Cl}$ (>99% and ~90%, respectively), but for peptide $\mathbf{1}^{2Cl}$ it would be deprotonated and negatively charged (only ~10% protonated). **(b)** Distributions of AGE adducts based on LC-MS analysis. Stacked bar graphs (left) are plotted as mean ± standard deviation for each mass adduct. Individual data points for this experiment performed at pH 7.3 can be found in Supplementary Figures 21.). Legend: blue, $[M+54]^A$; orange, $[M+54]^B$; gray, $[M+72]^A$; purple, $[M+72]^B$; green, [M+126]; lime, [M+80]; cyan, [M+144]. Data are derived from independent experiments: Left panel (pH 6.0): peptide **1** (n=2); $\mathbf{1}^{Cl}$ (n=2); $\mathbf{1}^{2Cl}$ (n=2). Center panel (pH 7.3): peptide **1** (n=8); $\mathbf{1}^{Cl}$ (n=3); $\mathbf{1}^{2Cl}$ (n=3). Right panel (pH 12.0): peptide **1** (n=2); $\mathbf{1}^{Cl}$ (n=2); $\mathbf{1}^{2Cl}$ (n=2). At pH 6 and 12, there are no statistically significant differences in total glycation or MGH-1 formation. However, at pH 7.3, there are significant differences in the total levels of glycation and resulting product distributions. We attribute this to two distinct phenomena: First, at neutral or acidic pH, MGH-1 formation requires the Tyr phenoxide to act as a base, thus a greater proportion of MGH-1 is observed for peptides $\mathbf{1}^{Cl}$ and $\mathbf{1}^{2Cl}$ as compared to peptide **1**. At pH 12, this trend disappears because the solution is basic enough for deprotonation to occur without any assistance from Tyr. Second, our published work and the work herein has demonstrated that clustered negative charge impedes glycation. This explains why peptide $\mathbf{1}^{2Cl}$, which has one extra negative charge as compared to peptides **1** or $\mathbf{1}^{Cl}$ at pH 7.3, exhibits lower levels of glycation overall. Additionally, this experiment supports our hypothesis that tyrosine is acting as a base, as MGH-1 levels increased with increasing pH. An ordinary one-way ANOVA using Dunnett's multiple comparison test, to compare each mean to the mean for peptide **1**, was used to determine if variants yield significantly different amounts of [M+54] (blue) or total glycation (black). p<0.05(*), p<0.01(**), p<0.001(***), p<0.0001(****). Additional statistical analysis is available in the Supplementary Data 1 file.

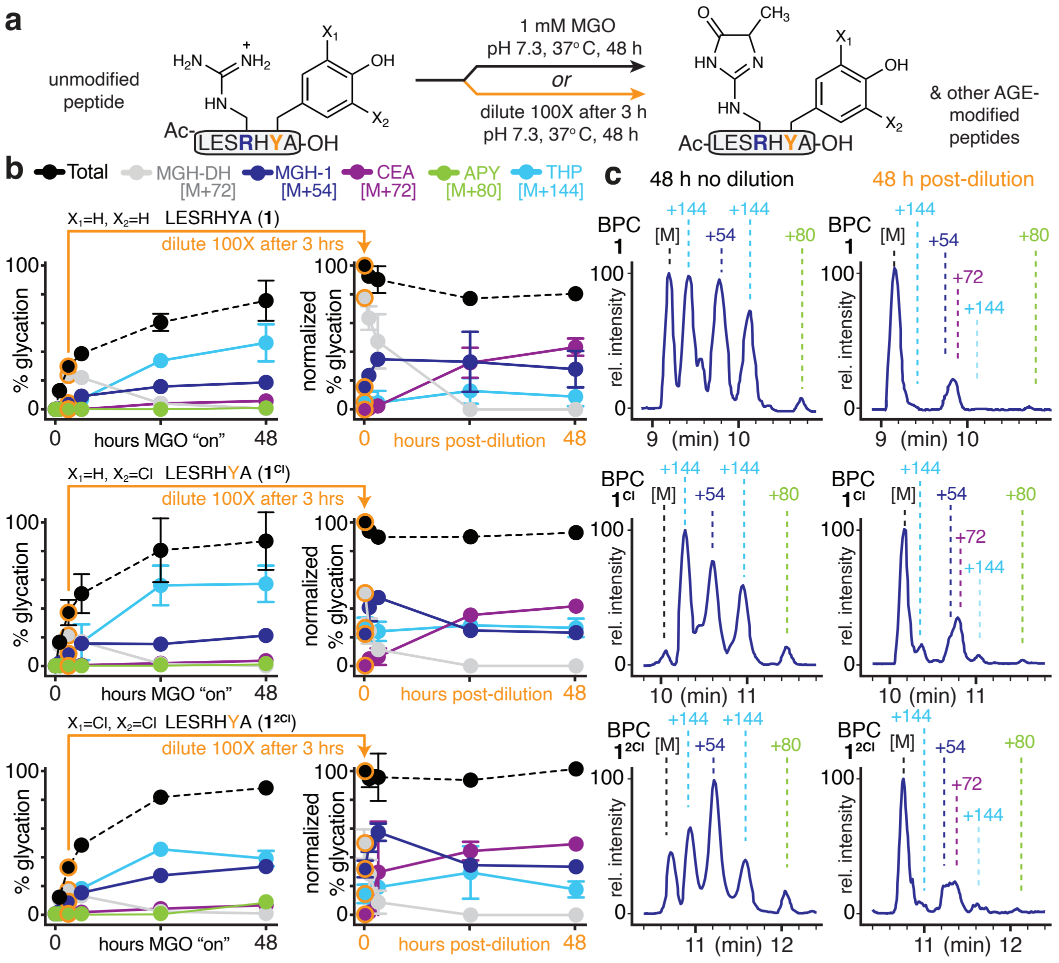

Supplementary Figure 23. Chlorinated Peptide 1 Variants Accelerate MGH-1 Formation. **(a)** Peptide **1** with either one (**1**^{Cl}) or two (**1**^{2Cl}) chloro-substitutions in the ortho-position to the phenolic hydroxyl of tyrosine were treated with MGO (1 mM, 1 equiv.) for up to 48 h, or diluted after 3 h and incubated for an additional 48 h (see **Main Text Figs. 4** & **6**). **(b)** AGE adducts (≥5% modified) were monitored over a 48 h period for peptides **1, 1**^{Cl} and **1**^{2Cl} in both undiluted and diluted time course studies. Legend: black, total glycation; blue, [M+54]^{A}; orange, [M+54]^{B}; gray, [M+72]^{A}; purple, [M+72]^{B}; green: [M+126]; lime, [M+80]; cyan, [M+144]. Data are plotted as mean values ± standard deviation and were derived from independent experiments, for LESRHYA (**1**): 1 h (n=3); 3 h (n=8); 6 h (n=3); 24 h (n=3); 48 h (n=3); 0 hpd (n=3); 1 hpd (n=3); 3 hpd (n=3) 24 hpd (n=3); 48 hpd (n=3); (**1**^{Cl}): 1 h (n=3); 3 h (n=3); 6 h (n=3); 24 h (n=3); 48 h (n=3); 0 hpd (n=3); 1 hpd (n=3); 3 hpd (n=3) 24 hpd (n=3); 48 hpd (n=3); **1**^{2Cl}: 1 h (n=2); 3 h (n=3); 6 h (n=2); 24 h (n=3); 48 h (n=3); 0 hpd (n=3); 1 hpd (n=3); 3 hpd (n=3) 24 hpd (n=3); 48 hpd (n=3). **(c)** Representative BPCs for the 48 h time point with no dilution (*left*), as well as the 48 hours post dilution (hpd) time point, after dilution (*right*), each display a 2 min retention time window starting approximately 0.2 min before elution of the parent peak [M]. The major species are parent peptide, [M]; two chromatographically resolved isomers of [M+144], a single [M+54] adduct (MGH-1), and, in peptide **1**^{Cl} and **1**^{2Cl}, an [M+80] adduct likely corresponding to argpyrimidine (APY). After 48 h of treatment with MGO (undiluted, *left*) LC-MS analysis reveals similarities in the distribution of AGEs formed for peptides **1, 1**^{Cl} and **1**^{2Cl}. After 48 h of incubation post dilution (*right*), there is even greater similarity in the AGE product distributions for these three peptides. We attribute this finding to the observation that upon dilution, reactivity is restricted solely to intramolecular reactions and/or rearrangements, rather than the addition of new equivalents of MGO. As a result, by 48 hpd peptides **1, 1**^{Cl} and **1**^{2Cl} all have similar product distributions. However, major differences are observed in the rate of AGE interconversion for these peptides (see **Main Text Fig. 6**). This finding suggests that chlorinated peptide **1** variants, with greater levels of phenoxide, accelerate the mechanistic steps that form MGH-1, but do not significantly alter product distributions.

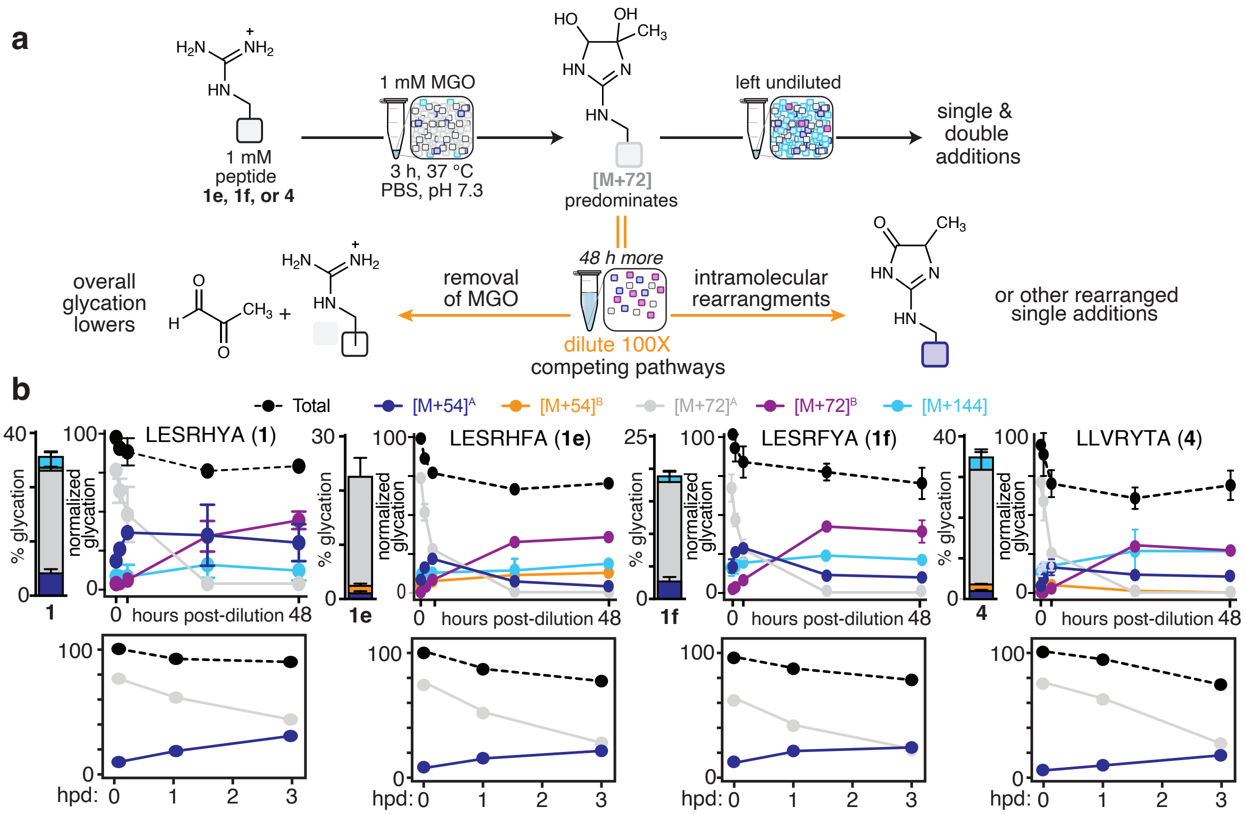

Supplementary Figure 24. Peptide Sequence Can Extend AGE Lifetimes. (a) Reactions were incubated with MGO (1 mM, 1 equiv.) for 3 hours and were then diluted 100X into phosphate buffer at the same pH. The resulting AGE product distributions were analyzed by LC-MS and quantified for **(b)** peptide **1,** peptide **1e**, peptide **1f** and peptide **4** (n≥3). Bar graphs to the left indicate the AGE product distribution and total amount of glycation observed at 3 h, prior to dilution. The dilution time course is normalized to the glycation observed at 3 h. Legend: black, total glycation; blue, $[M+54]^A$; orange, $[M+54]^B$; gray, $[M+72]^A$; purple, $[M+72]^B$; cyan, [M+144]. Data are plotted as mean values ± standard deviation and were derived from independent experiments: LESRHYA (**1**): 0 hpd (n=3); 1 hpd (n=3); 3 hpd (n=3) 24 hpd (n=3); 48 hpd (n=3); LESRHFA (**1e**): 0 hpd (n=3); 1 hpd (n=3); 3 hpd (n=3) 24 hpd (n=3); 48 hpd (n=3); LESRFYA (**1f**): 0 hpd (n=3); 1 hpd (n=2); 3 hpd (n=3) 24 hpd (n=2); 48 hpd (n=2); LLVRYTA (**4**): 0 hpd (n=3); 1 hpd (n=3); 3 hpd (n=3) 24 hpd (n=3); 48 hpd (n=4). Individual data points for the 3 h time point are shown in **Supplementary Figs. 8-10**. In general, all peptides led to similar patterns, as compared to peptide **1** (see **Main Text Figures 4**, **5** & **6**), however all with significantly less [M+54] adduct normalized to total glycation. For instance, each started with a decay of the $[M+72]^A$ product corresponding to MGH-DH. As observed for peptide **1**, upon dilution, MGH-DH can react further to form MGH-1 or may reverse to regenerate the parent peptide. In peptide **1**, after 48 h of dilution, approximately 19% of total observed glycation is lost, whereas in peptides **1e, 1f** & **4** there is a substantially higher loss (29.1%, 31.0%, and 27.1%, respectively). Because these latter peptides lack the ideal peptide 1 sequence that biases MGH-1 formation, they are more likely to form the kinetically favored MGH-3 (as seen in **1e** and **4**), which can be more readily hydrolyzed or reversed. This data supports our conclusions that the optimized peptide **1** scaffold partially contributes to glycation by extending the lifetimes of the AGE adducts that form (see also **Main Text Fig. 4, 5** & **6**).

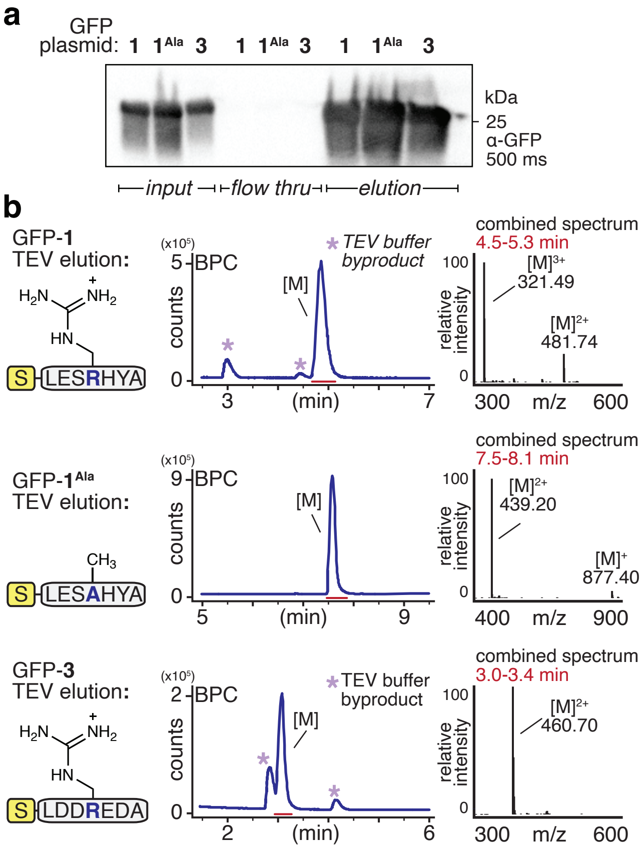

Supplementary Figure 25. Expression and Enrichment of GFP-1, GFP-1^{Ala}, and GFP-3 from HEK-293T cells. GFP proteins fused to a C-terminal peptide **1** (LESRHYA), **1**^{Ala} (LESAHYA), or **3** (LDDREDA) sequence were expressed in HEK-293T cells (see **Supplementary Methods**). **(a)** Following a GFP enrichment protocol, a representative western blot against GFP shows that not only is each construct expressed similarly in HEK-293T cells (lanes 1-3), but also that they are equivalently enriched (lanes 7-9) when eluted by SDS-PAGE to release the full-length construct from the resin. No differences were observed when MGO treatment steps were included (see also **Supplementary Fig. 26**), based on two replicate experiments. **(b)** Following TEV proteolysis to liberate only the C-terminal peptide fragments, the resulting peptide-containing eluates were analyzed by LC-MS. Representative BPCs and spectra for each major peak show each peptide was successfully identified after GFP enrichment and TEV treatment and were obtained with roughly similar counts, based on three replicate experiments. Please note that due to differences in retention time for the peptide **1**, **1**^{Ala}, or **3** fragments, the TEV buffer byproducts (*) are most notable in GFP-**1** and GFP-**3** elutions.

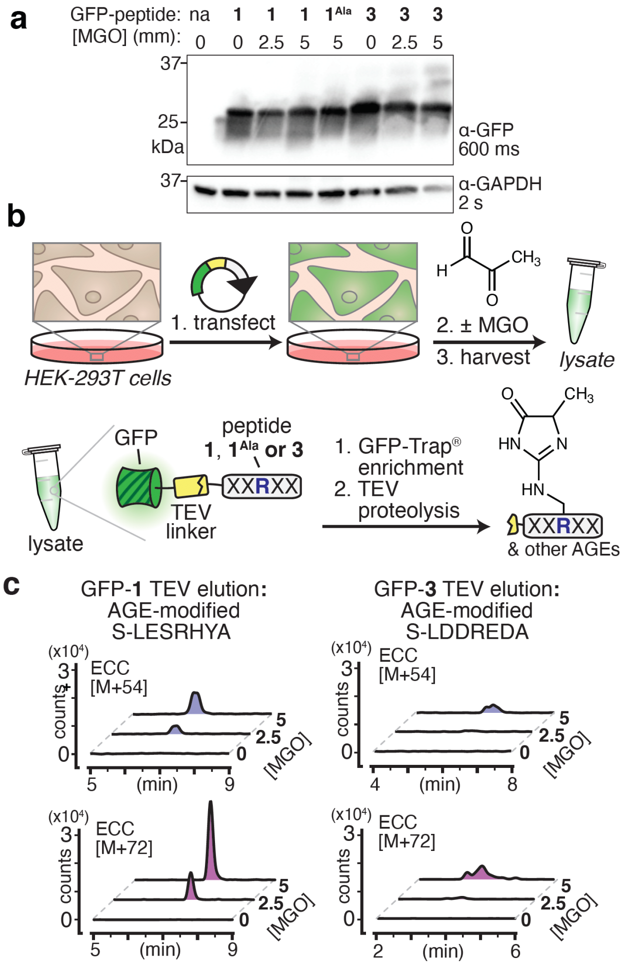

Supplementary Figure 26. MGO treatment of GFP-1, GFP-1^{Ala}, and GFP-3 in HEK-293T cells (a) A representative Western blot analysis, probing with α-GFP antibodies, shows that cells treated with increasing concentrations of MGO express GFP-**1,** GFP-**1^{Ala}**, and GFP-**3** proteins at comparable levels, based on two replicate experiments is shown. **(b)** As in **Main Text Fig. 7,** HEK-293T cells were seeded, transfected with plasmids encoding GFP-**1,** GFP-**1^{Ala},** and GFP-**3**, and were subsequently treated with 0 mM, 2.5 mM, or 5 mM MGO (see **Supplementary Methods**). After MGO treatment, cells were harvested and the resulting lysates were enriched by GFP immunoprecipitation, and subsequent TEV-mediated proteolysis to release the peptide of interest. **(c)** Following this treatment, GFP-**1** was found to exhibit more overall glycation than GFP-**3** at multiple MGO concentrations, as shown in the extracted compound chromatograms (ECCs) for the [M+54] and [M+72] adducts of the peptides released from GFP-**1** and GFP-**3**. These results confirm that the peptide **1** sequence leads to more [M+54] formation and more overall glycation than the peptide **3** sequence when fused to a full-length protein target expressed in mammalian cells. Thus, the findings reported herein can be expected to be directly applicable to glycation in cellular systems.

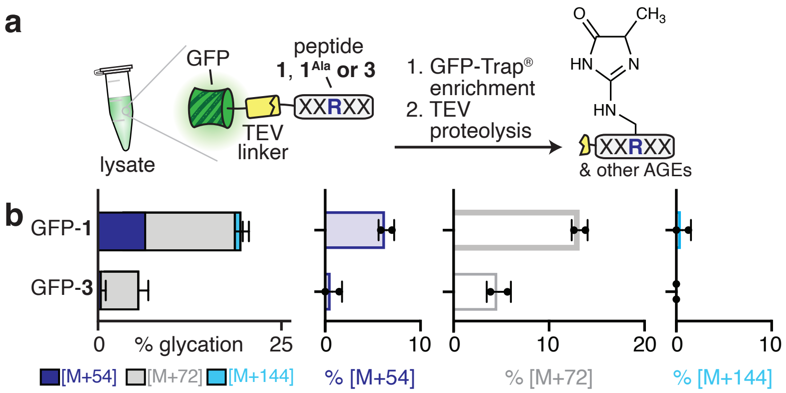

Supplementary Figure 27. Companion to Main Text Figure 7. The same data that appear in **Main Text Figure 7g** are replotted here to show individual data points. **(a)** Scheme depicting the glycation of these peptides on a full-length GFP-protein fusion expressed in cells, followed by enrichment and TEV proteolysis for analysis by mass spectrometry. **(b)** Distribution of AGEs observed for peptides **1**, **1**^{F}, **1**^{Cl} and **1**^{2Cl} after 3 h of MGO treatment. Data are plotted as mean values ± standard deviation and were derived from two independent replicate experiments. Legend: blue,[M+54]; gray, [M+72]; cyan, [M+144].

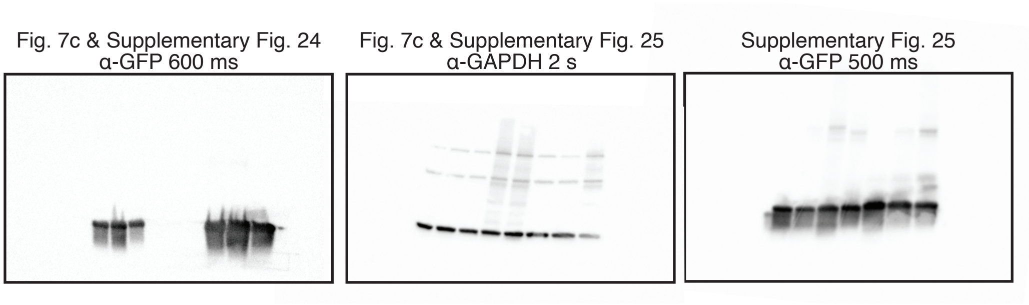

Supplementary Figure 28. Uncropped Immunoblots. Immunoblots are shown with chemiluminescence exposure times. Images were captured using a ChemiDoc Gel Imaging System (BioRad).

Supplementary References

(1) Oss, M.; Kruve, A.; Herodes, K.; Leito, I. Electrospray Ionization Efficiency Scale of Organic Compounds. *Anal. Chem.* **2010**, *82* (7), 2865–2872. https://doi.org/10.1021/ac902856t.

(2) Sjoblom, N. M.; Kelsey, M. M. G.; Scheck, R. A. A Systematic Study of Selective Protein Glycation. *Angewandte Chemie International Edition* **2018**, *57* (49), 16077–16082. https://doi.org/10.1002/anie.201810037.

(3) Ahmed, N.; Dobler, D.; Dean, M.; Thornalley, P. J. Peptide Mapping Identifies Hotspot Site of Modification in Human Serum Albumin by Methylglyoxal Involved in Ligand Binding and Esterase Activity. *J. Biol. Chem.* **2005**, *280* (7), 5724–5732. https://doi.org/10.1074/jbc.M410973200.

(4) Lam, K. S.; Lebl, M.; Krchňák, V. The "One-Bead-One-Compound" Combinatorial Library Method. *Chem. Rev.* **1997**, *97* (2), 411–448. https://doi.org/10.1021/cr9600114.

(5) Cai, W.; Gao, Q.-D.; Zhu, L.; Peppa, M.; He, C.; Vlassara, H. Oxidative Stress-Inducing Carbonyl Compounds from Common Foods: Novel Mediators of Cellular Dysfunction. *Mol Med* **2002**, *8* (7), 337–346.

(6) Wang, T.; Streeter, M. D.; Spiegel, D. A. Generation and Characterization of Antibodies against Arginine-Derived Advanced Glycation Endproducts. *Bioorganic & Medicinal Chemistry Letters* **2015**, *25* (21), 4881–4886. https://doi.org/10.1016/j.bmcl.2015.06.013.

(7) Thornalley, P. J. Dicarbonyl Intermediates in the Maillard Reaction. *Annals of the New York Academy of Sciences* **2005**, *1043* (1), 111–117. https://doi.org/10.1196/annals.1333.014.

(8) Rabbani, N.; Thornalley, P. J. Dicarbonyl Stress in Cell and Tissue Dysfunction Contributing to Ageing and Disease. *Biochemical and Biophysical Research Communications* **2015**, *458* (2), 221–226. https://doi.org/10.1016/j.bbrc.2015.01.140.

(9) Fritz, K. S.; Petersen, D. R. An Overview of the Chemistry and Biology of Reactive Aldehydes. *Free Radic Biol Med* **2013**, *59*, 85–91. https://doi.org/10.1016/j.freeradbiomed.2012.06.025.

(10) Bollong, M. J.; Lee, G.; Coukos, J. S.; Yun, H.; Zambaldo, C.; Chang, J. W.; Chin, E. N.; Ahmad, I.; Chatterjee, A. K.; Lairson, L. L.; Schultz, P. G.; Moellering, R. E. A Metabolite-Derived Protein Modification Integrates Glycolysis with KEAP1–NRF2 Signalling. *Nature* **2018**, *562* (7728), 600. https://doi.org/10.1038/s41586-018-0622-0.

(11) Harvey, D. J. Derivatization of Carbohydrates for Analysis by Chromatography; Electrophoresis and Mass Spectrometry. *Journal of Chromatography B* **2011**, *879* (17), 1196–1225. https://doi.org/10.1016/j.jchromb.2010.11.010.
