# Supplementary material for: Synergistic sequence contributions bias glycation outcomes
Source: Nat Commun. 2021 Jun 3;12:3316. doi: 10.1038/s41467-021-23625-8 (PMC8175500; doi:10.1038/s41467-021-23625-8)
Supplement: Supplementary file 3 — Description of Additional Supplementary Files [file 41467_2021_23625_MOESM3_ESM.docx]

Description of Additional Supplementary Files

Title: Supplementary Data 1

Description: The Supplementary Data 1 file provides further information about the statistical analysis that was performed, including exact p values for each analysis.
