# Supplementary material for: Synergistic sequence contributions bias glycation outcomes
Source: Nat Commun. 2021 Jun 3;12:3316. doi: 10.1038/s41467-021-23625-8 (PMC8175500; doi:10.1038/s41467-021-23625-8)
Supplement: Supplementary file 5 — Reporting Summary [file 41467_2021_23625_MOESM5_ESM.pdf]

nature research

Corresponding author(s): Rebecca Scheck

Last updated by author(s): Dec 16, 2020

Reporting Summary

Nature Research wishes to improve the reproducibility of the work that we publish. This form provides structure for consistency and transparency in reporting. For further information on Nature Research policies, see our Editorial Policies and the Editorial Policy Checklist.

Statistics

For all statistical analyses, confirm that the following items are present in the figure legend, table legend, main text, or Methods section.

n/a	Confirmed	
☐	☒	The exact sample size (*n*) for each experimental group/condition, given as a discrete number and unit of measurement
☐	☒	A statement on whether measurements were taken from distinct samples or whether the same sample was measured repeatedly
☐	☒	The statistical test(s) used AND whether they are one- or two-sided<br>*Only common tests should be described solely by name; describe more complex techniques in the Methods section.*
☐	☒	A description of all covariates tested
☐	☒	A description of any assumptions or corrections, such as tests of normality and adjustment for multiple comparisons
☐	☒	A full description of the statistical parameters including central tendency (e.g. means) or other basic estimates (e.g. regression coefficient) AND variation (e.g. standard deviation) or associated estimates of uncertainty (e.g. confidence intervals)
☐	☒	For null hypothesis testing, the test statistic (e.g. *F*, *t*, *r*) with confidence intervals, effect sizes, degrees of freedom and *P* value noted<br>*Give P values as exact values whenever suitable.*
☒	☐	For Bayesian analysis, information on the choice of priors and Markov chain Monte Carlo settings
☐	☒	For hierarchical and complex designs, identification of the appropriate level for tests and full reporting of outcomes
☒	☐	Estimates of effect sizes (e.g. Cohen's *d*, Pearson's *r*), indicating how they were calculated

Our web collection on statistics for biologists contains articles on many of the points above.

Software and code

Policy information about availability of computer code

Data collection	MS data were collected using Agilent MassHunter Data Acquisition Software Version B.05.01 and Bruker MicroFlex Acquisition Software. NMR data were recorded using Bruker TopSpin V2.1.
Data analysis	Data anlaysis was completed using the Agilent MassHunter Qualitative Analysis Version B.06.00, equipped with BioConfirm Software, PEAKS Studio V7.5, Bruker's FlexAnalysis Software, and TopSpin V2.1/V3.5. Microsoft Excel was used to calculate % modification. Prism 8 was used to plot data and perform statistical analyses. All figures were prepared using Adobe Illustrator.

For manuscripts utilizing custom algorithms or software that are central to the research but not yet described in published literature, software must be made available to editors and reviewers. We strongly encourage code deposition in a community repository (e.g. GitHub). See the Nature Research guidelines for submitting code & software for further information.

Data

Policy information about availability of data

All manuscripts must include a data availability statement. This statement should provide the following information, where applicable:

- Accession codes, unique identifiers, or web links for publicly available datasets
- A list of figures that have associated raw data
- A description of any restrictions on data availability

Processed numerical data used to generate bar and line graphs shown in Figures 2, 3, 4, 5, and 6 are available in a Supplemental Excel spreadsheet. Raw data in the form of LC-MS chromatograms or spectra appear in Main Text Figures 2, 3, 4 & 5, and Supplementary Figures 2, 3, 5, 8, 11, 13, 15, 16 & 18. An additional file with detailing all statistical analysis used in this study is also provided as a Supplemental Excel spreadsheet. DNA plasmid sequences used for the expression of peptide-fused GFP variants, as well us full, uncropped immunoblots are available in the Supplementary Information. Additional raw data are available upon request from the corresponding author.

Field-specific reporting

Please select the one below that is the best fit for your research. If you are not sure, read the appropriate sections before making your selection.

☒ Life sciences ☐ Behavioural & social sciences ☐ Ecological, evolutionary & environmental sciences

For a reference copy of the document with all sections, see nature.com/documents/nr-reporting-summary-flat.pdf

Life sciences study design

All studies must disclose on these points even when the disclosure is negative.

Sample size	All peptide experiments were replicated at least three times. In some cases, additional replicates were performed for the purposes of internal controls, or if there was inconsistent reproducibility between replicates. Cellular experiments were repeated at least two times using identical conditions. Detailed information about sample size is available in each figure caption.
Data exclusions	none
Replication	All replicate experiments were completed independently on different days.
Randomization	N/A
Blinding	N/A

Reporting for specific materials, systems and methods

We require information from authors about some types of materials, experimental systems and methods used in many studies. Here, indicate whether each material, system or method listed is relevant to your study. If you are not sure if a list item applies to your research, read the appropriate section before selecting a response.

Materials & experimental systems

n/a	Involved in the study	
☐	☒	Antibodies
☐	☒	Eukaryotic cell lines
☒	☐	Palaeontology and archaeology
☒	☐	Animals and other organisms
☒	☐	Human research participants
☒	☐	Clinical data
☒	☐	Dual use research of concern

Methods

n/a	Involved in the study	
☒	☐	ChIP-seq
☒	☐	Flow cytometry
☒	☐	MRI-based neuroimaging

Antibodies

Antibodies used	The anti-MGH antibody used is a mouse monoclonal antibody commercially available from Cell BioLabs, INC. (Cat# STA-011). The rat anti-GFP antibody (3H9) was purchased from Chromotek. Inc. (STA-011). The mouse anti-GAPDH primary antibody was purchased from MilliporeSigma (G8795). Alkaline phosphatase-conjugated goat anti-mouse secondary antibody was purchased from Abcam (AB7069). HRP-linked anti-mouse secondary antibody (7076V), and anti-rat secondary antibody (7077S) were purchased from Cell Signaling Technology.
Validation	The primary antibodies used in this study were each validated by the manufacturer and in some cases, subsequent follow up. Specifically, the anti-MGH antibody was developed and validated against MGH targets in the research lab of Dr. Helen Vlassara at the Division of Experimental Diabetes and Aging, Department of Geriatrics, Mount Sinai School of Medicine, New York, NY, USA. This work was published in the Journal of Molecular Medicine Volume 8, Number 7, July 2002. See also the Supporting Information for detailed protocols and additional referencing.

Eukaryotic cell lines

Policy information about cell lines

Cell line source(s)	HEK-293T, (ATCC® CRL-3216™)
Authentication	The ATCC validates their commercially-available cell lines using morphology, kyrotyping and PCR-based screening.
Mycoplasma contamination	Cell lines cultured in our lab are tested for mycoplasma every ~6 months and were found to be mycoplasma-free.

Commonly misidentified lines<br>(See ICLAC register)	N/A
